# Gender inequality and self-publication are common among academic editors

**DOI:** 10.1038/s41562-022-01498-1

**Published:** 2023-01-16

**Authors:** Fengyuan Liu, Petter Holme, Matteo Chiesa, Bedoor AlShebli, Talal Rahwan

**Affiliations:** 1 Computer Science, Science Division, New York University Abu Dhabi, Abu Dhabi, UAE; 2 Department of Computer Science, Aalto University, Espoo, Finland; 3 Center for Computational Social Science, Kobe University, Kobe, Japan; 4 Laboratory for Energy and Nano Science, Khalifa University of Science and Technology, Abu Dhabi, UAE; 5 Department of Physics and Technology, UiT - The Arctic University of Norway, Tromsø, Norway; 6 Social Science Division, New York University Abu Dhabi, Abu Dhabi, UAE

**Keywords:** Scientific community, Social sciences

## Abstract

Scientific editors shape the content of academic journals and set standards for their fields. Yet, the degree to which the gender makeup of editors reflects that of scientists, and the rate at which editors publish in their own journals, are not entirely understood. Here, we use algorithmic tools to infer the gender of 81,000 editors serving more than 1,000 journals and 15 disciplines over five decades. Only 26% of authors in our dataset are women, and we find even fewer women among editors (14%) and editors-in-chief (8%). Career length explains the gender gap among editors, but not editors-in-chief. Moreover, by analysing the publication records of 20,000 editors, we find that 12% publish at least one-fifth, and 6% publish at least one-third, of their papers in the journal they edit. Editors-in-chief tend to self-publish at a higher rate. Finally, compared with women, men have a higher increase in the rate at which they publish in a journal soon after becoming its editor.

## Abstract (web summary)

Using publication and editorial team composition records from more than 1,000 journals, Liu and coauthors uncover pervasive gender inequalities among academic editors. Only 8% of editors-in-chief are women. Nearly 6% of editors publish one-third of all their papers in the journal they edit, and this self-publication pattern is stronger among men editors.

## Main

A recurring theme in the study of science is the accumulative advantage that elites enjoy in academia, be it highly cited scientists^1,2^, prestigious institutions^3^ or influential countries^4^. Elites reinforce their status via scientific publications^1,5^, and editors play a key role in this process by having the final say about what gets published^6,7^, thereby controlling the channel through which scientists receive prestige and recognition^8^. Moreover, editors themselves are scientific elites, who have gained recognition from their community as experts in their fields^9,10^. Therefore, characterizing the appointment and publication patterns of editors is key to understanding scientific elites.

Unfortunately, not all scientists have an equal chance of becoming editors. Women, historically marginalized in academia, face additional barriers in attaining scientific opportunities in general^11–14^, and elite status in particular^15–19^. In this vein, there has been widespread, yet fragmented, evidence showing that women are underrepresented on editorial boards^20–28^. Gender diversity on editorial boards is not only important in its own right^29^, but also has broader implications. An inclusive editorial board signals that the journal is open to all authors^30^, implying that the underrepresentation of female editors may create a vicious cycle that further deters women from participating in science^29^.

While being the gatekeepers of science, editors also actively seek opportunities to publish. Although some editors are full-time professionals (for example, those handling journals such as *Cell*, *Nature* and *Science*), the vast majority of editors are research-active academics, who perform editorial duties in addition to being a scientist. Such editors would benefit from publishing original research articles, as the evaluation of academics heavily relies on bibliometric outcomes^1,31^. Sometimes editors publish their findings in the journals they edit^32–38^, occasionally resulting in controversies^39–42^. Such controversies are fuelled by the possibility that the editors’ submissions are treated favourably, which may be considered as ‘an abuse of the scientific publishing system’^41,42^.

Both the self-publishing behaviour of editors and the underrepresentation of women have received widespread attention in different disciplines; see Supplementary Tables 1 and 2 for a summary of this literature. Nevertheless, key aspects remain missing due to the lack of a longitudinal dataset that spans multiple disciplines. In particular, none of the studies compare the gender gap and self-publishing behaviour across disciplines, as they only focus on one discipline each. The only exceptions are the work of Mauleón et al.^10^ and Bošnjak et al.^34^, but their analyses are restricted to Spanish and Croatian journals, respectively. Another limitation in the literature is the lack of comparison between editors and other research-active scientists, with the exception of Mauleón et al.^10^, whose analysis is restricted to Spanish journals only. Such a comparison is critical, as it provides a discipline-specific, and year-specific, benchmark against which gender disparity and editor self-publishing can be measured.

In addition to the comparison between editors and research-active scientists, other comparisons can be informative when analysing the self-publishing behaviour of editors. First, by comparing editors with their colleagues—those who serve on the same editorial board—one would account for the culture of the journal in question, thereby detecting editors whose publication rate is high relative to the norm in that journal. Second, by comparing the publication patterns of the editors before and after the start of their editorship, one might detect whether there is a notable difference in the number of papers they publish in their journal right after assuming their editorial role. Third, by comparing female with male editors, one might determine whether there are marked gender differences in self-publication rates. These critical comparisons are absent from the literature, except for the works of Campanario^8^, Walters^38^ and Mani et al.^35^ The former two compared editors with other scientists, while the latter paper compared the publication pattern before and after one becomes an editor, but each of these studies considered only a single subdiscipline. As for any comparison between male and female editors, and between editors and their colleagues, these are completely absent from the literature.

Here, to fill the aforementioned gaps in the literature, we parsed more than 173,000 editorial pages from Elsevier—a publisher behind one-fifth of global research output, garnering one-quarter of citations worldwide^43^. This enabled us to extract information pertaining to 103,000 editors, including their affiliations, their disciplines, the names of the journals they edited and the years during which they served as editors. Collectively, these editors served on 85,000 issues of 1,167 Elsevier journals spanning 15 disciplines and multiple decades; see Supplementary Note 1 for details on how scientists’ disciplines were inferred, and Supplementary Table 3 for the distribution of editors across disciplines. Furthermore, following other studies in the literature^24,44–47^, we used a state-of-the-art classifier (Methods), allowing us to identify the gender of 81,000 editors and 4,700 editors-in-chief with high confidence; see Supplementary Table 4 for the distribution of those editors across disciplines. Finally, to retrieve the publication record of any given editor, we used Microsoft Academic Graph (MAG)—a dataset of 220 million publications and 240 million scientists^48,49^, which has been widely used in the Science of Science literature^44,47,50–55^. More specifically, we matched the editors in our dataset to the scientists in MAG based on their names, affiliations and disciplines, thereby identifying the publication records of 20,000 editors and 1,600 editors-in-chief who had a unique match in MAG; see Methods for more details on the data collection, and Supplementary Table 5 for the distribution of those editors across disciplines. The resulting datasets offer a unique opportunity to address the aforementioned shortcomings in the literature, and analyse editorial patterns at an unprecedented scale.

## Results

### Editor characteristics

First, we explore the characteristics of editors-to-be (here, we refer to editors in any role as ‘editors’) before the start of their editorship and compare them with an average scientist. To this end, for every editor, we randomly select a scientist whose discipline and academic birth year—the year when their first paper was published—match that of the editor. Then, we compare the pair in terms of citation count, paper count, h-index, collaborator count and affiliation rank. Note that the attributes of the editor are measured before their editorship starts, implying that the measurements are not influenced by the potential boost in visibility associated with being an editor. Moreover, the scientist being compared with the editor has their attributes measured in the same year, implying that the pair had the same career length when the measurements were taken. Finally, it should be noted that those scientists may themselves include editors of different publishing houses, as would be expected from the average scientist in MAG whom those scientists are meant to represent.

**Fig. 1:**
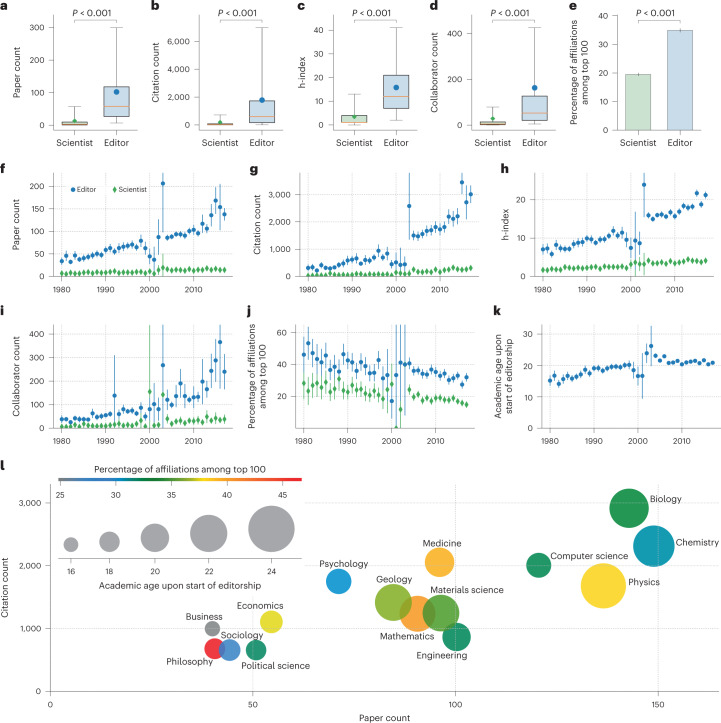
Editors’ characteristics upon the start of editorship. Each editor (*n* = 19,064) is compared with a randomly selected scientist whose discipline and first year of publication matches that of the editor; descriptive statistics are measured at the year preceding the start of the editorship, with error bars representing the 95% CI. **a**–**e**, Comparing editors with scientists in terms of paper count (**a**), citation count (**b**), h-index (**c**), collaborator count (**d**) and percentage of those whose affiliation ranks among the top 100 (**e**); circles and diamonds represent the sample means of editors and scientists, respectively; the boxes extend from the lower to upper quartile values of the data, with a line at the median; whiskers extend until the 5th and the 95th percentiles; *P* values are calculated using two-sided Welch’s T-tests (**a**–**d**) and two-sided Fisher’s exact tests (**e**); all *P* < 10^−250^. **f**–**j**, Comparing editors with scientists over time in terms of paper count (**f**), citation count (**g**), h-index (**h**), collaborator count (**i**) and percentage of those whose affiliation ranks among the top 100 (**j**). **k**, For each year, the mean academic age of editors upon the start of their editorship. **l**, Editors’ paper count (*x* axis), editors’ citation count (*y* axis), editors’ academic age (circle size) and percentage of editors whose affiliation ranks among the top 100 (circle colour) across disciplines; the differences in the circle sizes are exaggerated to improve visibility. Data are presented as mean ± 95% CI (**e**–**k**).

If editors are scientific elites, we would expect their bibliometric outcomes to be much higher than that of average scientists. Indeed, compared with an average scientist of the same academic age and in the same discipline, an editor tends to have seven times more papers (102 versus 13), eight times more citations (1,786 versus 193) and four times greater h-index (16 versus 3); see Fig. 1a–c. Note that these results disregard editorials; see Methods for more details on how editorials were identified. As for the number of collaborators, an editor has on average 163 at the start of the editorship, while the average scientist has about 29 (Fig. 1d). In terms of affiliation, 35% of editors are affiliated with a top-ranked institution—one that is ranked among the top 100 according to the Academic Ranking of World Universities^56^—compared with just 20% for scientists (Fig. 1e). Supplementary Fig. 1 shows the distribution of the data in Fig. 1a–d, while Supplementary Figs. 2–6 show a breakdown of Fig. 1a–e over disciplines. Moreover, instead of sampling a single scientist per editor, we sample 50 and 200 with replacement, and find broadly similar trends (see Supplementary Figs. 7 and 8).

Next, we analyse how the characteristics of editors upon the start of their editorship have changed over the past four decades. Specifically, let (*e*,*j*) denote an editor–journal pair such that editor *e* served on journal *j*. Moreover, let year ${}_{1}^{(e,j)}$ be the first year of the editorship, and let year ${}_{0}^{(e,j)}$ be the year that precedes it. Then, for any given year *y* ∈ [1980, 2017], we consider every *e*,*j* such that year ${}_{0}^{(e,j)}=y$, and measure the characteristics of *e* and their matched scientists at the year *y*. The results are depicted in Fig. 1f–k. As can be seen, the expected number of citations that an editor has accumulated by the start of their editorship has increased almost tenfold over the past decades (from 311 in 1980 to 3,014 in 2017), the number of accumulated papers has more than quadrupled (from 34 to 138), the h-index has tripled (from 7 to 21), the number of collaborators has increased more than sixfold (from 38 to 240), while the percentage of those affiliated with top-ranked institutions has decreased (from 46% to 32%). Next, we examine the gap between editors and scientists over the past decades. Comparing 1980 with 2017, we find that the gap in productivity has increased almost fivefold (from 27 in 1980 to 124 in 2017), the gap in impact has increased more than ninefold (from 289 to 2706), the gap in h-index has more than tripled (from 5 to 17), while the gap in collaborator count has increased more than sixfold (from 32 to 202). As for the percentage of those affiliated with a top-ranked institution, it has decreased over the years for both editors and scientists at about the same rate (from 46% to 32% for editors, and from 28% to 15% for scientists), suggesting that this trend is not related to changes in the way editors are recruited, but rather to changes in the global demographics in academia. Again, these results remain unchanged when sampling 50 and 200 scientists per editor. Finally, looking at the academic age of the editors upon the start of their editorship, we find that it has increased from 15 years in 1980 to about 20 years in 2017 (Fig. 1k). These findings suggest that, when it comes to assuming an editorial role, being impactful, productive, connected and experienced seem to matter more than being affiliated with a top-ranked institution. This seems to persist even when excluding Biology—the discipline with the highest h-indices, citation count, collaborator count and editor count—as shown in Supplementary Fig. 9. Note that in Fig. 1f–k an anomaly can be seen around the years 1998–2003. Upon enquiry, Elsevier representatives clarified that this anomaly is an artefact of an incomplete capture of all articles during the first years of their transition from print to online.

Having analysed how different characteristics of editors change over time, we now compare those characteristics across disciplines. More specifically, Fig. 1l compares editors from different disciplines in terms of the number of citations and papers that an editor has accumulated, as well as their affiliation rank and academic age, upon the start of the editorship. We find that Biology recruits the most highly cited editors, with 2,900 citations on average, while Chemistry recruits the most productive editors, with an average of 149 papers. In contrast, the impact seems to matter the least when recruiting editors in Philosophy, Sociology and Political Science, while productivity seems to matter the least when recruiting editors in Business and Philosophy. As for academic age, we find that Business recruits the youngest editors, with 16 years of experience on average, while Physics recruits the eldest, with 24 years of experience. We calculate the average academic age of editors across disciplines, and find it to be just over 20 years. Finally, in all disciplines, the percentage of editors affiliated with a top-ranked institution ranges from 25% to 47%, with Philosophy having the greatest percentage.

### Gender composition of editors

**Fig. 2:**
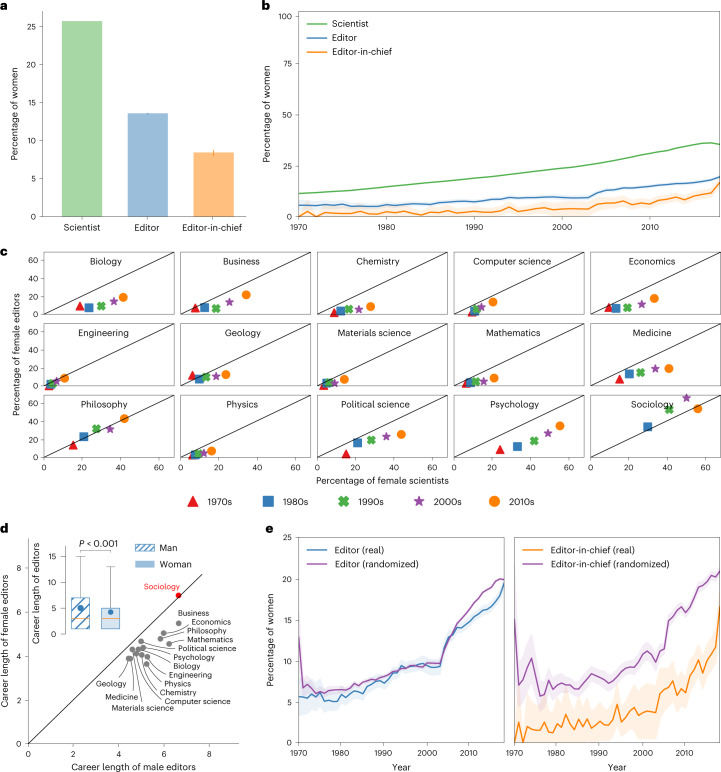
Gender disparity in editorship. **a**, Percentage of women among scientists (*n* = 42,831,834), editors (*n* = 80,776) and editors-in-chief (*n* = 4,692). **b**, Percentage of women among scientists, editors and editors-in-chief over time. **c**, Percentage of female editors against percentage of female scientists across disciplines in the 1970s, 1980s, 1990s, 2000s and 2010s. **d**, Average editorial career length of female (*n* = 12,644) versus male editors (*n* = 68,132) overall (inset, with circles and diamonds represent the sample mean of man and woman, respectively; the boxes extend from the lower to upper quartile values of the data, with a line at the median; whiskers extend until the 5th and the 95th percentiles) and across disciplines (scatter plot); red highlights the discipline in which the career length of female editors is greater than that of male editors; *P* values are calculated using two-sided Welch’s T-test (inset); the exact *P* value is 1.46 × 10^–50^. **e**, Percentage of women among editors and editors-in-chief in real versus randomized data over time. Error bars and shaded regions represent 95% CI. Data are presented as mean ± 95% CI (**a**, **b** and **e**).

We conclude our exploratory analysis by studying gender disparity among editors. As can be seen in Fig. 2a, although women are already underrepresented among scientists (26% of all unique scientists in MAG), they are even more underrepresented among editors and editors-in-chief (14% and 8%, respectively). Moreover, the gap remained stable over the past five decades; the proportion of female editors has consistently remained around half that of female scientists, although gender parity has been steadily increasing in science in general (Fig. 2b). For example, in 2017, women represented 36% of scientists, but only 18% of editors; these proportions are extremely similar to those in 1970, when women represented 11.3% of scientists and 5.7% of editors. As for female editors-in-chief, their proportion has remained consistently smaller than that of female editors since 1970. These broad trends seem to persist when excluding Biology—the discipline with the highest h-indices, citation count, collaborator count and editor count—as shown in Supplementary Fig. 10.

Let us now examine the gender disparity across disciplines. Figure 2c depicts the proportion of female editors against that of female scientists across disciplines during the 1970s, 1980s, 1990s, 2000s and 2010s. Apart from Sociology, the proportion of female scientists in any given discipline has remained greater than the proportion of female editors in that discipline; see how the vast majority of shapes fall under the diagonal. Similar patterns are observed for editors-in-chief (Supplementary Fig. 11). To obtain a better understanding of this phenomenon, we analysed the length of editorial careers, that is, the number of years during which editors assume their role. The box plot in Fig. 2d compares the average editorial career length of women versus men, while the scatter plot compares these quantities across disciplines. As can be seen, the editorial career length of men (mean 5.03; 95% confidence interval (CI) 4.99 to 5.08) is greater than that of women (mean 4.24; 95% CI 4.17 to 4.32; *t*_80,774_ = 15.02, *P* < 0.001, *β* = 0.15, 95% CI 0.13 to 0.16); this holds across all disciplines except Sociology. Supplementary Fig. 12 shows similar patterns for editors-in-chief (mean of men 3.93; 95% CI 3.78 to 4.07; mean of women 2.62; 95% CI 2.41 to 2.84; *t*_4,685_ = 6.27, *P* < 0.001, *β* = 0.28, 95% CI 0.22 to 0.34), with the editorial career length of male editors-in-chief being greater than that of their female counterparts in all disciplines except Engineering, Geology and Materials Science.

As we have shown thus far, women have been consistently underrepresented on editorial boards across disciplines over the past decades. Let us now investigate whether this phenomenon can be explained by gender differences in productivity, impact and career lengths, or whether additional hidden factors are at play. To this end, we use a randomized baseline model whereby each editor (or editor-in-chief) is replaced with a randomly chosen scientist who may have a different gender but is identical in terms of discipline and academic age (just like the matched scientists in Fig. 1), and similar in terms of productivity and impact (both binned into deciles). In this model, the randomly selected scientist replaces the original editor for the entire duration of his/her editorial career. Such a null model simulates a world where the editors in each discipline are recruited solely based on their experience and research output while completely disregarding their gender. We generated 50 such worlds and computed the average percentages of female editors and editors-in-chief therein. It should be noted that such analysis cannot be done using any of the datasets previously considered in the literature, as it requires the publication records of not only the editors but also all research-active scientists in any given discipline. The results of this analysis are depicted in Fig. 2e. As can be seen in the left panel, the representation of women among editors in a randomized world exhibits similar trends to those observed in the real world. This suggests that the gender gap among editors can be explained by the lack of women with sufficiently high productivity and impact, which, in turn, can be explained by attrition of women from academia^47^. In contrast, looking at the right panel of Fig. 2e, we find a clear and persistent gap between the real and counterfactual worlds in terms of the proportion of female editors-in-chief. This suggests that factors other than career length, productivity and impact may be at play, and these factors seem to persist over the past five decades.

### Editors’ self-publishing behaviour

**Fig. 3:**
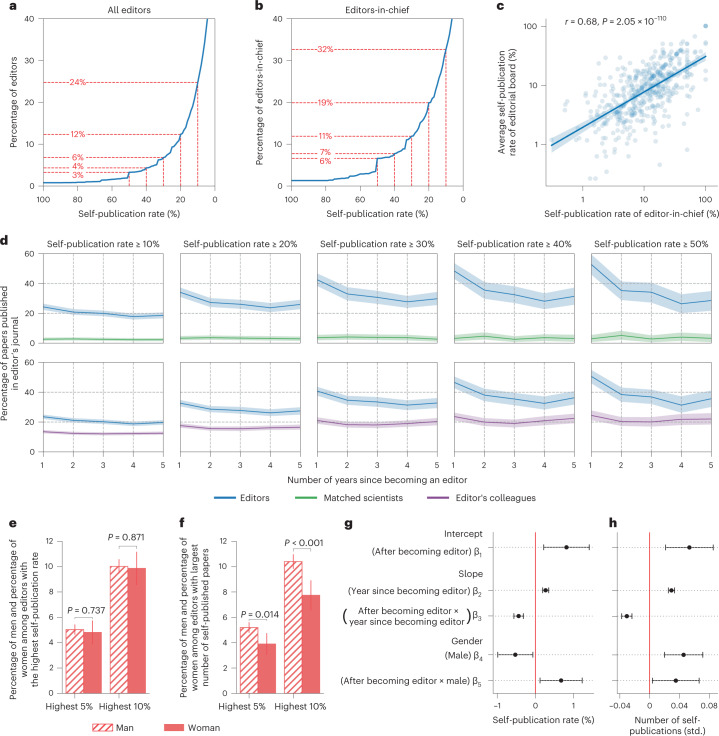
Self-publication rates. **a**, Cumulative distribution of editors’ self-publication rate, highlighting the proportion of those whose rate is ≥ 0%, ≥ 20%, …, ≥ 50%. **b**, The same as (**a**) but for editors-in-chief. **c**, Correlation between the self-publication rates of editors-in-chief and their editorial boards; *r* represents the two-sided Pearson correlation coefficient, while the shaded region represents 95% CI of the regression estimate. **d**, Comparing editors whose self-publication rate is ≥ 10%, ≥ 20%, …, ≥ 50% to their matched scientists (upper row) and to their colleagues (lower row) in terms of the percentage of papers published in the editor’s journal. **e**, Out of all men and women, the percentage of those who fall among the top 5% and 10% of editors with the highest self-publication rates; *P* values are calculated using two-sided Fisher’s exact tests, *n*_male_ = 11,017, *n*_female_ = 1,978. **f**, The same as (**e**), but for those who have the largest number of papers published in their own journal (the exact *P* value for highest 10% is 2.08e-4). **g**, Regression-estimated temporal trend of the self-publication rate during the 5 years before, and the 5 years after, one becomes an editor; see Supplementary information for model specifications. **h**, The same as (**g**), but the outcome is the number of self-published papers, measured in terms of standard deviations (std.). Data are presented as mean ± 95% CI in (**d**) through (**h**).

Having analysed the gender disparity in editorial boards, we now shift our attention to another interesting aspect of editorship—the fact that some editors publish original research in the journal they edit. To reiterate, editorials are consistently excluded throughout this study; see Methods for details on how editorials are identified. We start off by analysing the editors’ self-publication rate—the percentage of their papers published in their own journal—during the 5-year period following the start of their editorship. Based on this, as well as the fact that the publication records we extract from MAG do not go beyond 2018, we restrict this analysis to the 12,995 editors who start editing their respective journals no later than 2014. For editors who quit before completing 5 years, the self-publication rate is measured only over the years during which they serve as editors, rather than over the full 5-year period following the start of their editorship. Let us start by examining the cumulative distribution of self-publication rates. We find 24% of editors publish at least one-tenth of their papers in the journal they edit (Fig. 3a). There is also a considerable percentage of editors who publish at least one-fifth of their papers (12% of editors) or even one-third of their papers (6% of editors) in their own journal. Among editors-in-chief, these percentages are even higher (Fig. 3b). More specifically, 32% of editors-chief publish at least one-tenth of their papers in the journal they edit, 19% self-publish at least one-fifth of their papers and 11% self-publish one-third of their papers. For a breakdown of these distributions across disciplines, see Supplementary Figs. 13 and 14. Next, we examine the correlation between the self-publication rate of the editors-in-chief and their editorial board (Fig. 3c). To improve the visualization, the data points are plotted on a log-log scale while omitting zero values; see Supplementary Fig. 15 for a linear-scale version of this plot. As can be seen, there is a significant positive correlation between the self-publication rate of the editor-in-chief and that of the editorial board, suggesting that the two are linked.

To better understand these patterns, for every *e*,*j* pair we compare *e* with randomly selected scientists who are not editors of *j* but are similar to *e* in terms of gender, discipline, rank of first affiliation and years during which they are research-active. Additionally, we ensure that *e* and their matched scientists are similar in terms of the rate at which they publish in *j* up to year ${}_{0}^{(e,j)}$. Note that this matching process is different from the two matching processes used earlier in Figs. 1 and 2e; see Methods for more details. Also note that the matched scientists may themselves be editors of other journals. As such, the outcome of this analysis reflects the difference between those who edit *j* and those who do not, rather than the difference between editors and non-editors. The results of this analysis are depicted in the upper row of Fig. 3d. As shown in this figure, regardless of the rate at which *e* publishes in *j*, there is a marked gap between *e* and their matched scientists. This observation suggests that the difference in the rate at which *e* and their matched scientists publish in *j* is associated with *e* becoming an editor of *j*, bearing in mind that both of them published in *j* at comparable rates before year ${}_{0}^{(e,j)}$ as shown in Supplementary Fig. 16 and Supplementary Table 6. A possible explanation for the observed increase in publication rate is the journal’s culture, whereby editors are expected to contribute papers as part of their editorial duties. To determine whether this is the case, for every editor *e* whose self-publication rate is ≥ 10%, ≥ 20%, …, ≥ 50%, we compare the self-publication rate of *e* to that of the average editor serving at the same time on the same editorial board. This comparison considers the years after, but not before, *e* becomes an editor, as these are the years during which the publication rate of *e* in *j* could be influenced by the journal culture. As shown in the bottom row of Fig. 3d, regardless of *e*’s self-publication rate, they publish in *j* at a greater rate than their average colleague.

Finally, we check whether there exist gender differences in terms of self-publication rate, as well as the number of self-published papers. To this end, we first calculate the percentage of men, and the percentage of women, who fall among the top 1%, 2%, …, 10% of editors with the highest self-publication rates (Fig. 3e and Supplementary Fig. 17a). We find that men and women are equally likely to be found among those with the highest self-publication rates (top 5%: 5.03% male, 4.8% female, *P* = 0.737; top 10%: 10.02% male, 9.86% female, *P* = 0.871, Fisher’s exact test). In other words, the gender composition of those with very high self-publication rates roughly reflects that of all editors. However, if we calculate the percentage of men, and the percentage of women, who fall among the top 1%, 2%, …, 10% of editors with the largest number of self-published papers (Fig. 3f and Supplementary Fig. 17b), we find more men among those with the highest numbers of self-published papers, and this difference is statistically significant (top 5%: 5.19% male, 3.89% female, *P* = 0.014; top 10%: 10.4% male, 7.74% female, *P* < 0.001, Fisher’s exact test). In other words, while men account for 84.8% of all editors, they account for 88.2% of the top 10% editors with the highest number of self-published papers.

To further investigate the gender differences in self-publication behaviour, we introduce a regression model to estimate the temporal trend of an editor’s self-publication rate each year, while allowing a structural break of the trend to happen around the time that one becomes an editor. Additionally, the regression model controls for gender as well as journal fixed effects (Fig. 3g and Supplementary Table 7). The model indicates that the self-publication patterns of editors exhibit statistically significant discontinuity around the time when the editorship starts, as both the intercept and the slope change significantly, providing further evidence of the link between becoming an editor of a journal and increasing the rate at which one publishes in that journal. The model also indicates that, around the start of the editorship, male editors show a higher increase in their self-publication rates compared with female editors. We repeat the same regression analysis but change the outcome to be the number of self-published papers per annum (Fig. 3h and Supplementary Table 8). Again, the model shows that the self-publication patterns of editors exhibit significant discontinuity around the time when the editorship starts, and male editors have a higher increase in the number of self-published papers after becoming editors.

**Fig. 4:**
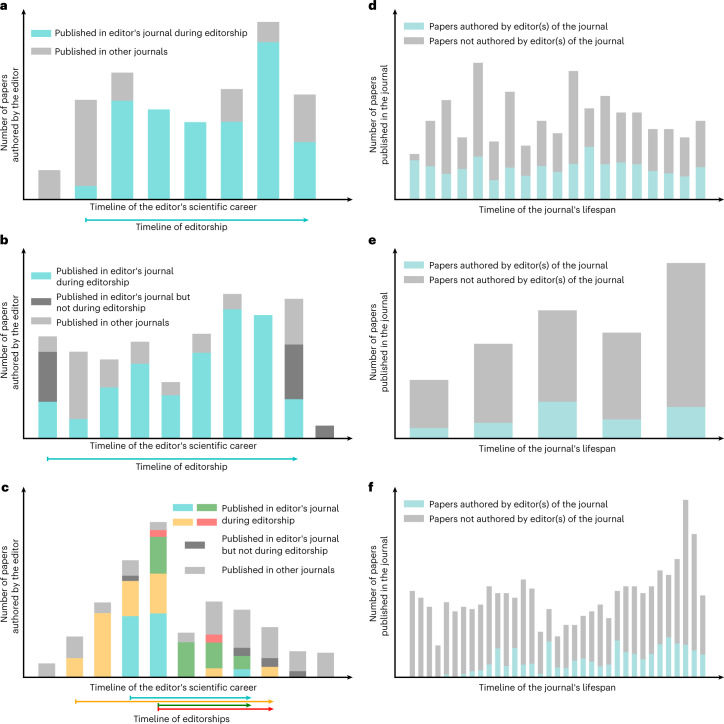
Extreme editors and extreme editorial boards. To preserve anonymity, ticks on the *x* axis and *y* axis are hidden and Gaussian noise is added to the bar heights. Out of all editors who publish at least 30 papers throughout their careers, subfigures (**a**), (**b**) and (**c**) correspond to the editor with the highest, second highest and third highest number of self-publications, respectively. For each of these editors, we show the total number of papers they publish as well as how many of those papers are published in the editor’s journal(s); results are aggregated over 5-year periods to preserve anonymity. The horizontal line(s) underneath the plot represent the span of the editorship(s). Out of all journals that have at least 30 papers, subfigures (**d**), (**e**) and (**f**) depict the journal with the highest, second highest and third highest proportion of papers whose authors include an editor of that journal, respectively.

To understand the limits of the above phenomenon, that is, the extent to which editors self-publish while continuing to serve on the editorial board, we identified the 15 editors who publish the highest percentage of papers in their journals during editorship. For each of them, we plotted the number of papers published per year throughout their scientific careers aggregated over 5-year periods, highlighting in different colours the proportion of the papers published in the journal(s) they were editing; see Fig. 4a–c for the three most extreme editors, and Supplementary Fig. 18 for the remaining 12. In these figures, random perturbations are added to the counts to preserve anonymity. Focusing on the most extreme editors, out of all the papers they published throughout their career, 72%, 66% and 65% were in their own journal(s) while they were serving as editors. These cases demonstrate that even if an editor publishes three-quarters of their entire career output in their own journal, they may continue to serve as editors for several decades. Similar trends were observed when considering the 15 (rather than the three) most extreme editors; see Supplementary Fig. 18. It is worth mentioning that 14 out of those editors are men, suggesting that women are less likely to engage in such extreme behaviour. Also noteworthy is the fact that 6 out of the 15 extreme editors are, in fact, editors-in-chief.

Having analysed extreme editors, let us now focus on the three extreme editorial boards corresponding to the journals with the highest percentage of papers authored by their editors. The results are depicted in Fig. 4d–f, which follow a similar layout compared with our previous analysis of extreme editors. Starting with the most extreme journal (Fig. 4d), one-third (35%) of the papers published therein have an active editor among the authors. As for the second and third most extreme journals (Fig. 4e,f), one-fifth (about 20%) of the papers published therein include authors who happen to be active editors. These cases demonstrate that editorial board members can author a substantial share of the papers published in the journal, and continue to do so for several decades.

## Discussion

Despite efforts to increase women’s representation on editorial boards^57^, the present findings reveal a persistent gender gap. Using an unprecedented dataset, our study contributes to the literature in two ways. First, we were able to examine the gender distribution among scientists and editors over the past five decades, revealing that the proportion of female editors persisted at about half that of female scientists, and that the proportion of female editors-in-chief has consistently been even smaller. Second, we were able to compare the gender gap across 15 disciplines, revealing that women have been consistently underrepresented among editors and editors-in-chief in every discipline other than Sociology. Furthermore, while gender disparity has often been measured in terms of impact^58,59^, productivity^58^ and career length^47^, we showed that, at least for editors-in-chief, gender disparity goes beyond what is predicted by these numbers, indicating a systematic role for non-meritocratic factors in the selection of editors-in-chief. This resonates with the past findings that women face a ‘glass ceiling’ in their professional careers^60^, and suggests that women face additional obstacles in being recognized as elite scientists in their respective disciplines. Overall, this study contributes to the literature advocating a more inclusive editorial board in particular, and a more inclusive scientific community in general^29,61^.

We also showed that a substantial amount of editors publish in the journal they edit, and provided the first comparison, to our knowledge, of self-publication behaviour across disciplines and genders. As such, our study contributes to the line of research exploring gender differences in academia^62–65^. Moreover, our unique dataset allowed us to understand how far editors can reach with their self-publication practice. Naturally, these findings raise the question: How much self-publication should be considered too much? Of course, there are perfectly innocuous explanations of why editors self-publish. Some may conduct research in a niche field with only a few alternative journals to publish in; others may be established scientists who self-publish their best works to boost the reputation of a young journal. Still, if there is anything that can be learned from recent scandals involving editors^39,40,66,67^, it is that the power enjoyed by editors can be exploited. For instance, consider those editors-in-chief who self-publish at high rates, despite being responsible for overseeing the review process of every submission, including their own. To an external observer, it may not be entirely clear how such articles are handled to circumvent the apparent conflict of interest. By providing an overview of the status quo of self-publishing practice, our study contributes to the discussion of whether self-publications should be governed with more transparency.

Our study is not without limitations. First, our work comes with the inherent restrictions of observational studies. In particular, although we use standard techniques such as matching and randomized baseline models to further our understanding of gender inequality and self-publication patterns, it is hard to pinpoint the underlying mechanisms behind these findings; this constitutes a potential direction for future research. Second, all analyses are done using editor data collected from Elsevier. Although this is the largest publisher in academia, other publishers could also be explored, which is left to future studies. Lastly, to infer gender at scale, the only practical solution was to use algorithmic tools. Despite their advantages, such tools are not 100% accurate. Although we restricted our analysis to names that are classified with at least 90% accuracy throughout the study, manual classification is likely to be more accurate.

There is more to the story of scientific publishing than statistics. Behind the numbers, some editors stand up for a more transparent selection of papers, and actively recruit board members from underrepresented groups, while others exploit their power to benefit their careers. After all, editors are humans. Our expectation of human behaviour in imperfectly transparent institutions determines the narrative: Is it reassuring that the majority of editors hardly self-publish? Or is it striking that 11% of editors-in-chief publish at least one-third of their papers in the journal they edit? Should we be satisfied with the increasing proportion of female editors over the past decades? Or should we be concerned that, despite all efforts to promote gender equality, women are still underrepresented among editors in nearly all disciplines? Either way, we hope our study, and the future work it may inspire, will contribute to a fairer, more transparent and more inclusive culture of scientific editorship.

## Methods

### Data

#### Data collection

Elsevier published 4,289 different journals in 2019, all of which are listed on ScienceDirect—a website operated by the publisher^68^. Each journal curates some or all of its past issues, and all of the articles that appeared in every curated issue. In addition to research articles, many journals list their editors on the Editorial Board page, which can be found in the first volume of each issue. These pages, which constitute the primary source of our editor-related data, were retrieved using the Elsevier Article Retrieval API^69^. In total, we collected 173,434 editorial board pages from 1,893 different journals. From these pages, we were able to extract the following information about each journal: title, issue, volume, discipline, publication date, editors’ names, editors’ affiliations and whether or not any given editor is an editor-in-chief. To retrieve the publication records of these editors, we paired them with scientists from the MAG dataset. In particular, an editor in Elsevier and a scientist in MAG are considered to be the same person if, and only if, they uniquely share the same name and affiliation. For details on how the name disambiguation problem is addressed in this study, see Supplementary Note 2. For any *e*,*j* pair, the first (last) year of editorship is assumed to be the publication year of the first (last) issue of *j* in which *e* is mentioned as an editor. Moreover, the editorial career of *e* (as an editor of *j*) is assumed to span the period between the first and last years of editorship (inclusive), implying that any gap years (if they exist) are included in our analysis. Similarly, the academic career of any scientist *s* is assumed to span the period between the publication years of their first and last papers. As a result, the academic age of *s* in any given year *y* is $y-{\,{{\mbox{y}}}{\mathrm{ear}}}_{{{\mbox{first}}}\,}^{s}+1$, where year ${}_{\,{{\mbox{first}}}\,}^{s}$ is the publication year of the first paper of *s*.

Editorials were then excluded from the publication record of each editor, to ensure that it consisted of scientific papers. To this end, we queried ScienceDirect to identify the type of each publication in Elsevier, and excluded over 13,000 publications falling under the following types: Book review, Conference info, Editorial, Encyclopedia, Erratum, News, Practice guideline and Product review. This left us with about 168,000 publications (co)authored by the 20,000 editors identified in MAG. Out of those publications, we randomly sampled 200 and manually verified that only two were, in fact, editorial pieces. Additionally, we manually examined all publications (co)authored by the three extreme editors considered in Fig. 4, and again found that only two were editorial pieces. This analysis suggests that our approach of identifying and excluding editorial pieces, while not perfect, is highly accurate.

#### Gender identification

Several gender classifiers have been proposed to date^58,70,71^. Following other studies in the literature^24,44–47^, we use *Genderize.io*, which has been shown to outperform other alternatives^70^. This classifier integrates publicly available census statistics to build a name database, mapping names to binary gender labels. In our gender-related analysis, we only considered scientists whose first names were classified with a confidence of ≥ 90%.

#### Dataset evaluation

In our gender-based analysis, we considered a dataset consisting of the 81,000 editors whose gender has been identified by *Genderize.io* with confidence of ≥ 90%; let us denote this dataset by *E*_gender_. On the other hand, when analysing the publication patterns of editors, we considered a dataset consisting of the 20,000 editors who had a unique matching entry in MAG, denoted by *E*_MAG_. These two datasets are likely to exhibit biases as they were not randomly sampled from all 103,000 Elsevier editors, denoted by *E*_all_. In this section, we aim to understand how these biases could affect our main findings.

We start off by comparing *E*_gender_ to *E*_all_ in terms of the relative size of each discipline. We found that the two are highly correlated (*r* = 0.99); see Supplementary Fig. 19a. High correlation could also be seen when comparing *E*_MAG_ with *E*_all_ (*r* = 0.79); see Supplementary Fig. 19b. Nevertheless, some disciplines are underrepresented compared with *E*_all_, while others are overrepresented. As a result, the observed proportion of women in *E*_gender_ may differ from that in *E*_all_, and the observed proportion of editors in *E*_MAG_ whose self-publications rates are ≥ 10%, …, ≥ 50% may also differ from that in *E*_all_. As shown in Supplementary Fig. 19a, Medicine is overrepresented in *E*_gender_, which could affect our gender-based findings, especially as Medicine amounts to more than a fifth of all editors in *E*_gender_. For instance, if this discipline happens to have fewer female editors than average, then our estimation of the gender gap (which is based on *E*_gender_) would be an overestimation of the overall gender gap (that is, the one in *E*_all_). To estimate the gender gap in *E*_all_, we multiplied the proportion of women in each discipline in *E*_gender_ by the size of that discipline in *E*_all_. Similarly, to estimate the percentage of editors whose self-publications rates are ≥ 10%, …, ≥ 50% in *E*_all_, we multiplied their proportion in each discipline in *E*_MAG_ by the size of that discipline in *E*_all_. As a result, the proportion of female editors becomes 12.95% (instead of the originally estimated 13.53%), and the percentage of editors whose self-publication rate is ≥ 10%, ≥ 20%, ≥ 30%, ≥ 40% and ≥ 50% becomes 27.21%, 13.79%, 7.71%, 4.88% and 3.66%, respectively (instead of 24.79%, 12.34%, 6.79%, 4.33% and 3.27%, respectively). This suggests that the situation may be grimmer than originally estimated, as the gender gap seems to be larger, and self-publication seems to be more widespread, once we adjust for differences in discipline size.

Next, we examine how representative *E*_MAG_ is of *E*_all_ in terms of the editors’ publication patterns. As there are no available datasets that provide the publication profiles of all 103,000 editors in *E*_all_, a practical alternative is to compare *E*_MAG_ with a random sample of *E*_all_, after manually identifying the publication profile of each sampled editor. Unfortunately, this approach also comes with its own limitations, as many editors do not have an online presence, making it extremely challenging, if not impossible, to manually identify their publication profile. With these limitations in mind, we sampled 500 editors from *E*_all_ and were able to manually identify the MAG entry of 264 of them using information available online; the set of those 264 editors is denoted by *E*_manual_. Then, we compared *E*_MAG_ with *E*_manual_ in terms of the confounders examined earlier in Fig. 1, namely: paper count, citation count, collaborator count, h-index, academic age and percentage of those affiliated with a top 100 institution. We found significant differences between *E*_MAG_ and *E*_manual_ in terms of paper count, collaborator count, h-index and percentage of editors whose affiliation is among the top 100; see Supplementary Fig. 20. These differences may lead to a biased estimation of the percentage of each type of editor. As a sensitivity analysis, we re-sampled the editors in *E*_MAG_ so that the distributions of all confounders are similar to *E*_manual_. After re-sampling, we found the percentage of editors whose self-publication rate is ≥ 10%, ≥ 20%, ≥ 30%, ≥ 40% and ≥ 50% becomes 24.23%, 12.72%, 7.61%, 5.02% and 4.04%, respectively (instead of 24.79%, 12.34%, 6.79%, 4.33% and 3.27%, respectively), and there is no significant difference between *E*_MAG_ and *E*_manual_ in terms of those percentages using two-sided Fisher’s exact tests. This analysis suggests that the differences in the above confounders do not bias the estimation of the percentage of editors with high self-publication rates. Note that the aforementioned re-sampling is only done as a part of this sensitivity analysis, and does not affect any other result in our study.

### Matching editors to scientists

In three parts of our study, we used matching techniques to compare editors against scientists with comparable attributes. This section provides detailed descriptions of each matching process.

First, in our descriptive analysis (Fig. 1), we aim to compare editors with average scientists who work in the same discipline and have the same academic age. Based on this, for each *e*,*j* pair, we compare *e* to scientists whose discipline and academic birth year—the year when their first paper was published—are the same as that of *e*.

Second, as part of our analysis of gender disparity, we apply a matching technique (Fig. 2e) to test the hypothesis that the underrepresentation of female editors can be explained by their lower publication and citation rates. To this end, for any *e*,*j* pair we randomly sample 50 scientists whose discipline and academic birth year are the same as *e*, and whose productivity and impact by the end of year ${}_{0}^{(e,j)}$ are comparable with *e* (that is, they fall within the same decile computed over each discipline in the given year).

Third, in our analysis of self-publication patterns, we apply a matching technique (top row of Fig. 3f) to analyse the editors whose self-publication rate is higher than a certain percentage. Here, the hypothesis is that the observed self-publication rate of an editor *e* in their own journal *j* can be explained by characteristics of *e* that are unrelated to *e* becoming an editor. To this end, given an *e*,*j* pair, we match *e* to a scientist *s* who is not an editor of *j* based on a number of confounders, including the rate at which they publish in *j*. Ideally, the rate of *e* and *s* should be similar up to year ${}_{0}^{(e,j)}$ (this way, if their rate starts to diverge after year ${}_{0}^{(e,j)}$, it suggests that the divergence is related to *e* becoming an editor of *j*). However, to increase the likelihood of finding a match for *e*, we do not require the rate of *s* to match that of *e* in year ${}_{0}^{(e,j)}$, but rather in a year *y* such that $| {\,{{\mbox{year}}}\,}_{0}^{(e,j)}-y| \le 3$. More specifically, we say that *e* matches *s* in year *y* if all of the following conditions are met:

- *e* and *s* have the same discipline.
- *e* and *s* have the same gender; for details on how gender is identified, see Gender identification.
- The rank of any first known affiliations of *e* and *s* fall in the same bin. Here, affiliations are ranked based on the 2019 Academic Ranking of World Universities (also known as the ‘Shanghai ranking’^56^), and are divided into the following bins: [1, 20]; [21, 50]; [51, 100]; [101, 300]; [301, 600]; [601, 999]; [1000, *∞*].
- The publication year of *e*’s first paper does not differ from that of *s* by more than 3 years.
- There exists a year, $y\in [{\,{{\mbox{year}}}}_{0}^{(e,j)}-3,{{{\mbox{year}}}\,}_{0}^{(e,j)}+3]$ such that:
  - The academic age of *e* in year${}_{0}^{(e,j)}$ does not differ from that of *s* in *y* by more than 10%.
  - The percentage of papers that *e* published in *j* in year${}_{0}^{(e,j)}$ does not differ from that of *s* in *y* by more than 10%.
  - The percentage of papers that *e* published in *j* up to year${}_{0}^{(e,j)}$ does not differ from that of *s* in *y* by more than 10%.

### Reporting summary

Further information on research design is available in the Nature Portfolio Reporting Summary linked to this article.

## Supplementary information

Supplementary Information Supplementary Figs. 1–20, Tables 1–8 and Notes 1–2.

Reporting Summary

## Data Availability

Our editors’ dataset was collected from Elsevier’s ScienceDirect database. A formal agreement between us and Elsevier mandates that data copied from the subscribed products cannot be provided to third parties in any substantial or systematic manner. However, for transparency reasons, we provide a sample set of 10 editors, which can be used to test the code for data collection and analysis, along with anonymized data for reproducing figures, all the while ensuring that our agreement with Elsevier is not breached. As for our publications’ dataset, that is, the Microsoft Academic Graph, a copy of it is available at https://zenodo.org/record/2628216#.Y4i9mnbP2Ul (ref. 72); a small subset of MAG that is sufficient to test our code is also provided. To retrieve the aforementioned datasets, visit the following link: https://github.com/Michael98Liu/fair-and-inclusive-scientific-publishing/tree/main/data (ref. 73). If you would like to obtain permission to collect and analyse data provided by Elsevier, please contact their customer consultant at k.bevilacqua@elsevier.com.

## Code availability

The Python code used to collect and clean the editors’ dataset, as well as the code used in the analysis and visualizations, are all freely available to download from https://github.com/Michael98Liu/fair-and-inclusive-scientific-publishing.
