# Supplementary material for: Gender inequality and self-publication are common among academic editors
Source: Nat Hum Behav. 2023 Jan 16;7(3):353–64. doi: 10.1038/s41562-022-01498-1 (PMC10038799; doi:10.1038/s41562-022-01498-1)
Supplement: Supplementary file 1 — Supplementary Figs. 1–20, Tables 1–8 and Notes 1–2. [file 41562_2022_1498_MOESM1_ESM.pdf]

nature human behaviour

Article

https://doi.org/10.1038/s41562-022-01498-1

Gender inequality and self-publication are common among academic editors

In the format provided by the authors and unedited

Contents

Supplementary Figures **2**

Supplementary Tables **22**

Supplementary Note 1: Inferring the Discipline of Authors and Journals **31**

Supplementary Note 2: Author Name Disambiguation **32**

Supplementary Figures

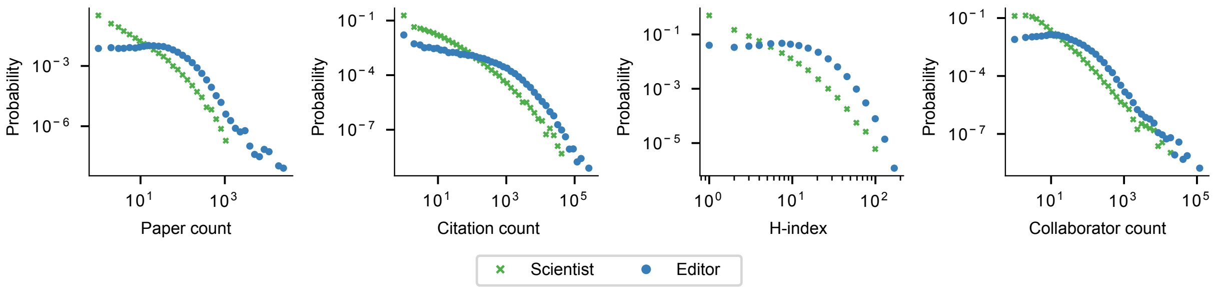

Supplementary Figure 1: **The distribution of the paper count, citation count, h-index, and collaborator count of editors and scientists.** Only 2 editors never published a single paper before the start of their editorship.

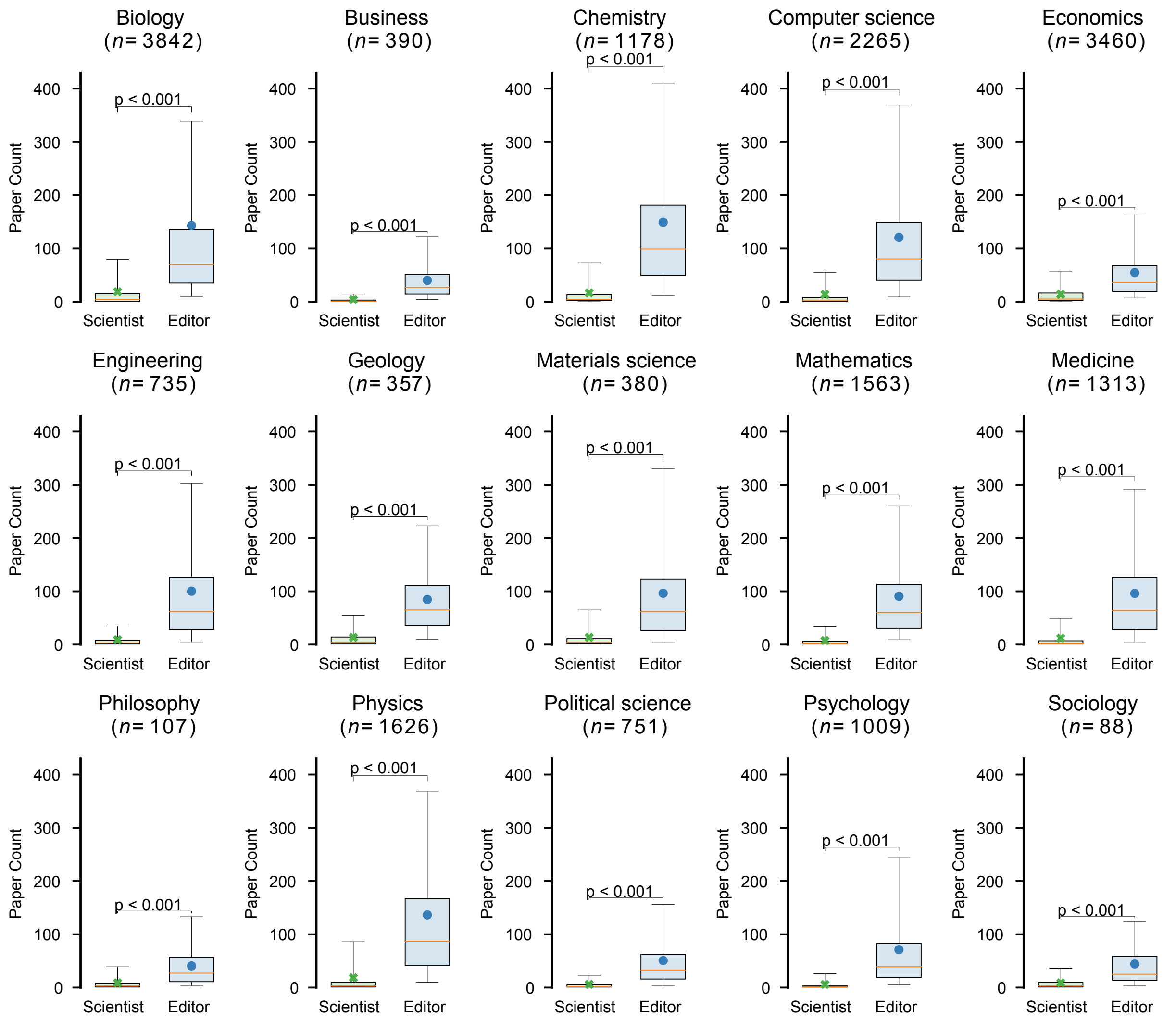

Supplementary Figure 2: **Paper count of editors upon the start of editorship across disciplines.** Each editor is compared to randomly selected scientists whose first year of publication matches that of the editor. The number of papers is measured at the year that precedes the start of the editorship. Diamonds and circles denote the population mean of scientists and editors, respectively; the boxes extend from the lower to upper quartile values of the data, with a line at the median; whiskers extend until the 5-th and the 95-th percentile. p-values are calculated using two-sided Welch's T-tests. All exact p-values are less than 10^{-250}. Sample size in each discipline is denoted in the title of each panel.

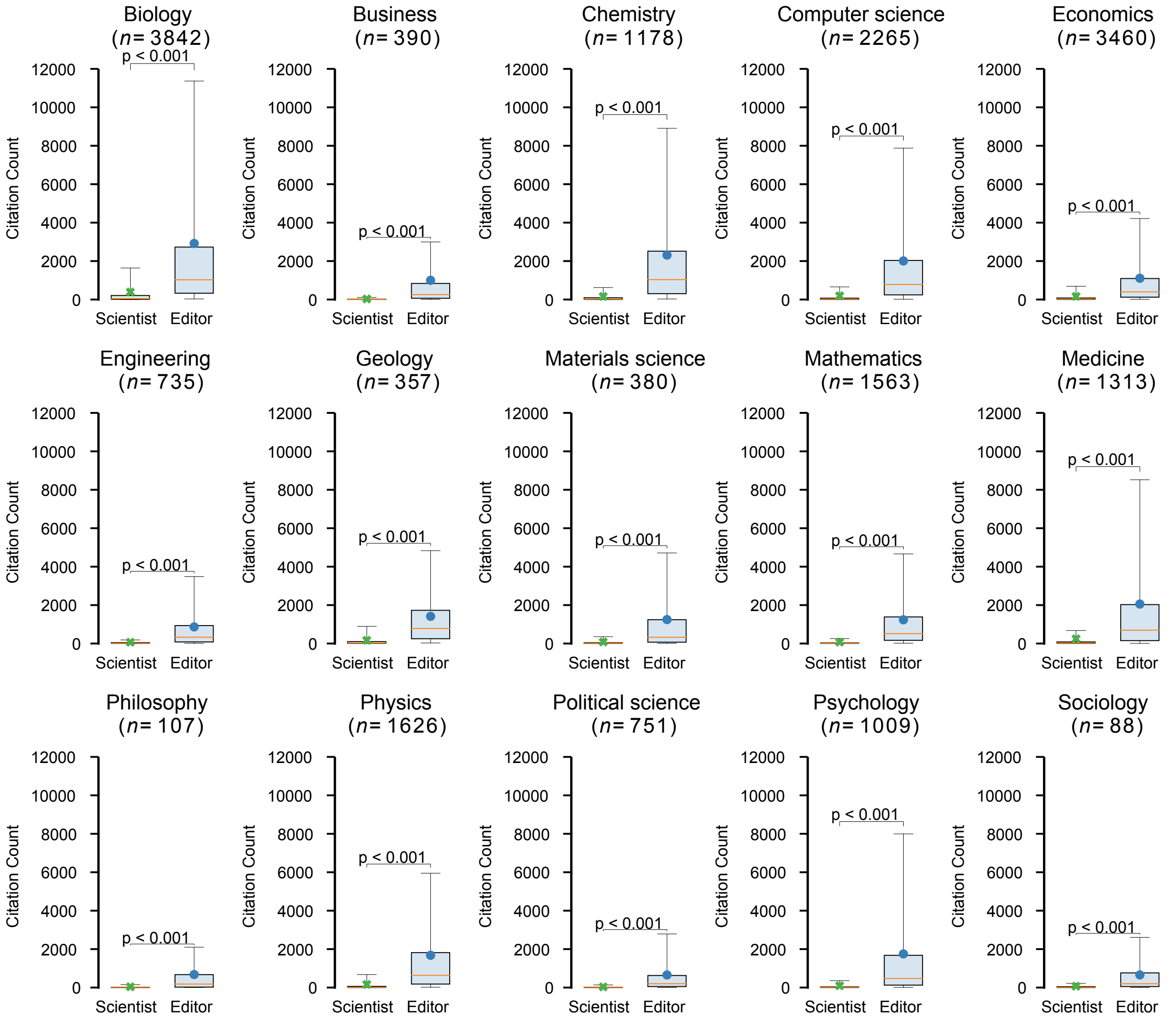

Supplementary Figure 3: **Citation count of editors upon the start of editorship across disciplines.** Each editor is compared to randomly selected scientists whose first year of publication matches that of the editor. The number of citations is measured at the year that precedes the start of the editorship. Diamonds and circles denote the population mean of scientists and editors, respectively; the boxes extend from the lower to upper quartile values of the data, with a line at the median; whiskers extend until the 5-th and the 95-th percentile. p-values are calculated using two-sided Welch's T-tests. Sample size in each discipline is denoted in the title of each panel.

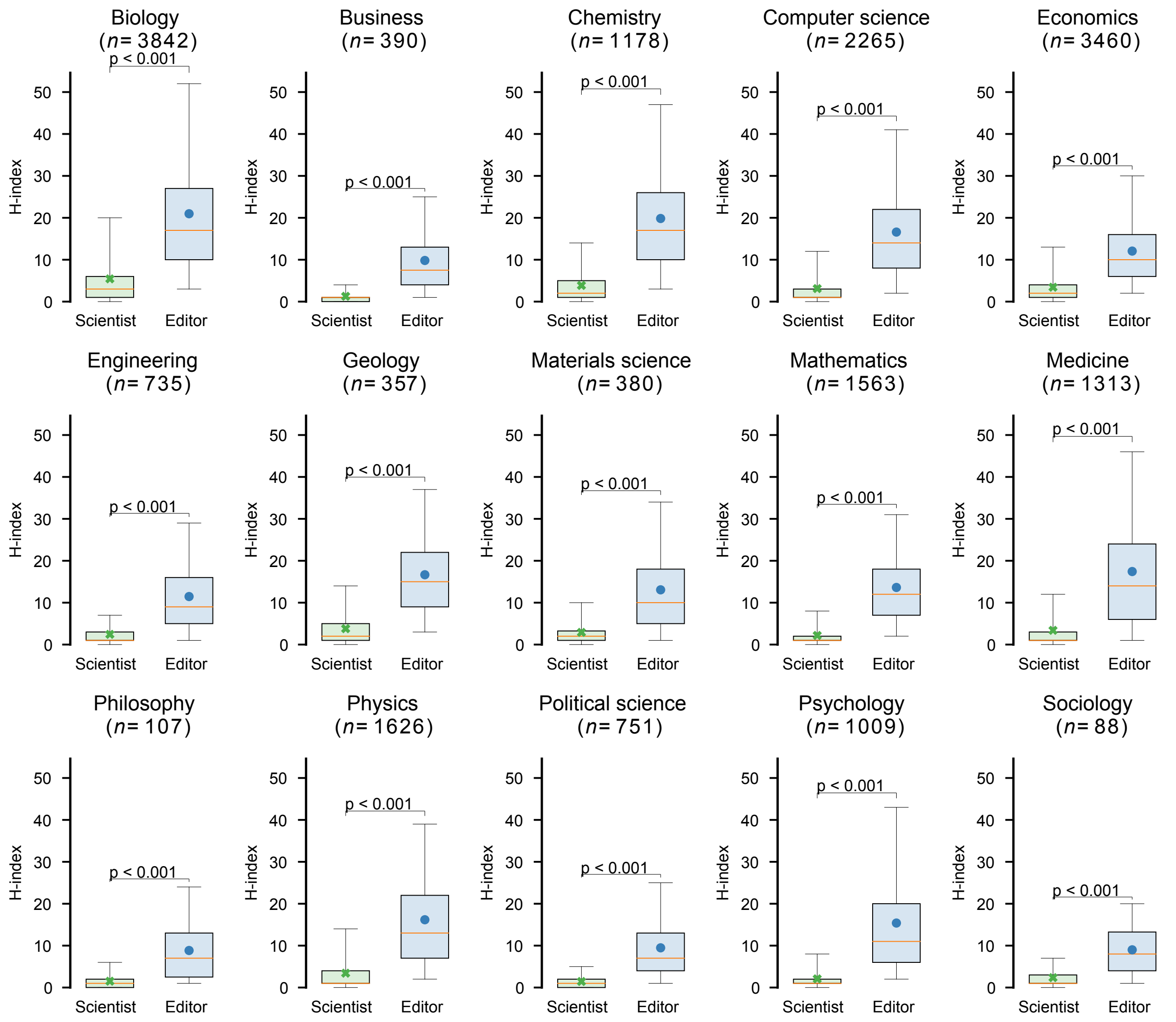

Supplementary Figure 4: **H-index of editors upon the start of editorship across disciplines.** Each editor is compared to randomly selected scientists whose first year of publication matches that of the editor. The h-index is measured at the year that precedes the start of the editorship. Diamonds and circles denote the population mean of scientists and editors, respectively; the boxes extend from the lower to upper quartile values of the data, with a line at the median; whiskers extend until the 5-th and the 95-th percentile. p-values are calculated using two-sided Welch's T-tests. All exact p-values are less than 10^{-250}. Sample size in each discipline is denoted in the title of each panel.

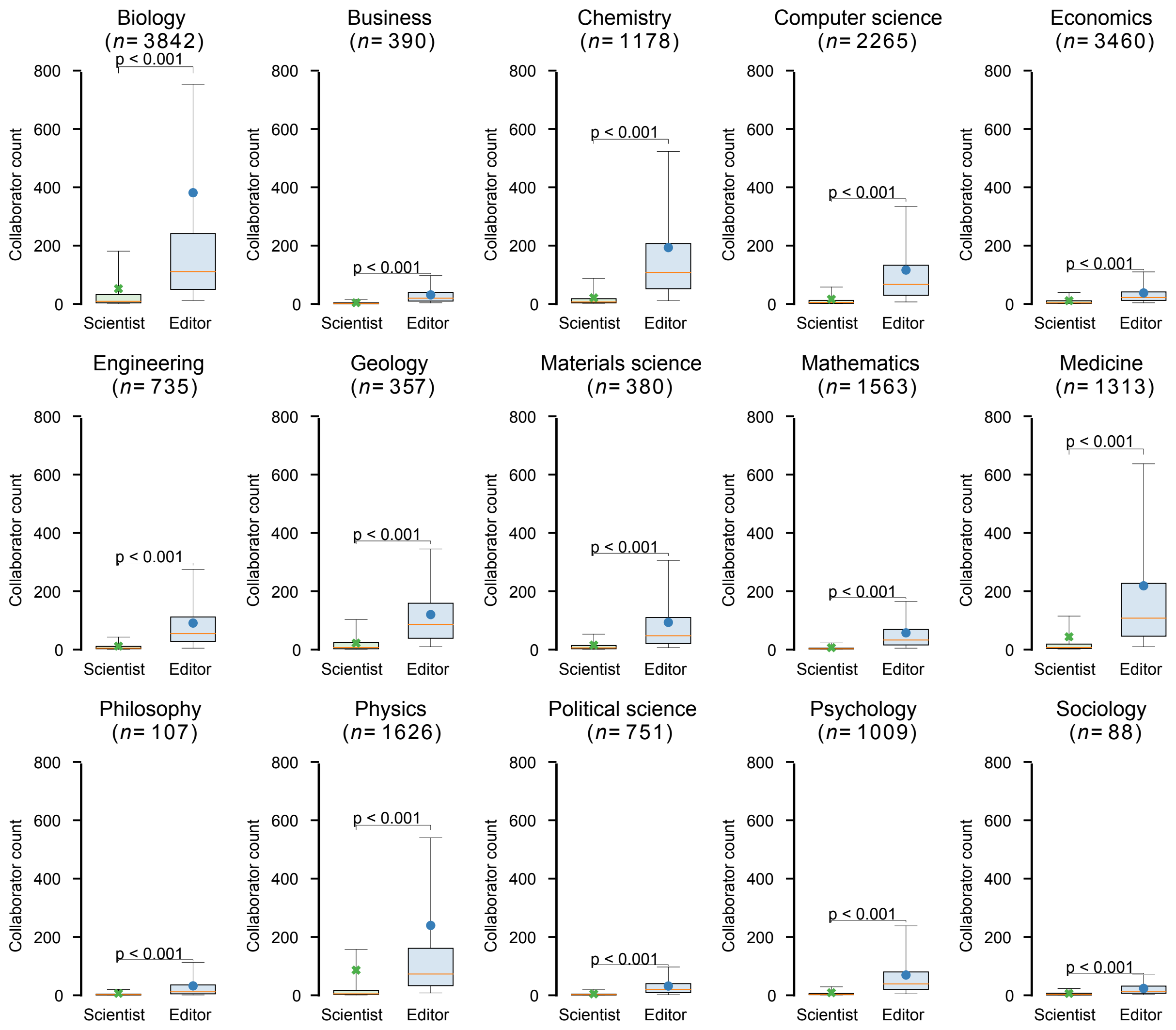

Supplementary Figure 5: **Collaborator count of editors upon the start of editorship across disciplines.** Each editor is compared to randomly selected scientists whose first year of publication matches that of the editor. The number of collaborators is measured at the year that precedes the start of the editorship. Diamonds and circles denote the population mean of scientists and editors, respectively; the boxes extend from the lower to upper quartile values of the data, with a line at the median; whiskers extend until the 5-th and the 95-th percentile. p-values are calculated using two-sided Welch's T-tests. All exact p-values are less than 10^{-250}. Sample size in each discipline is denoted in the title of each panel.

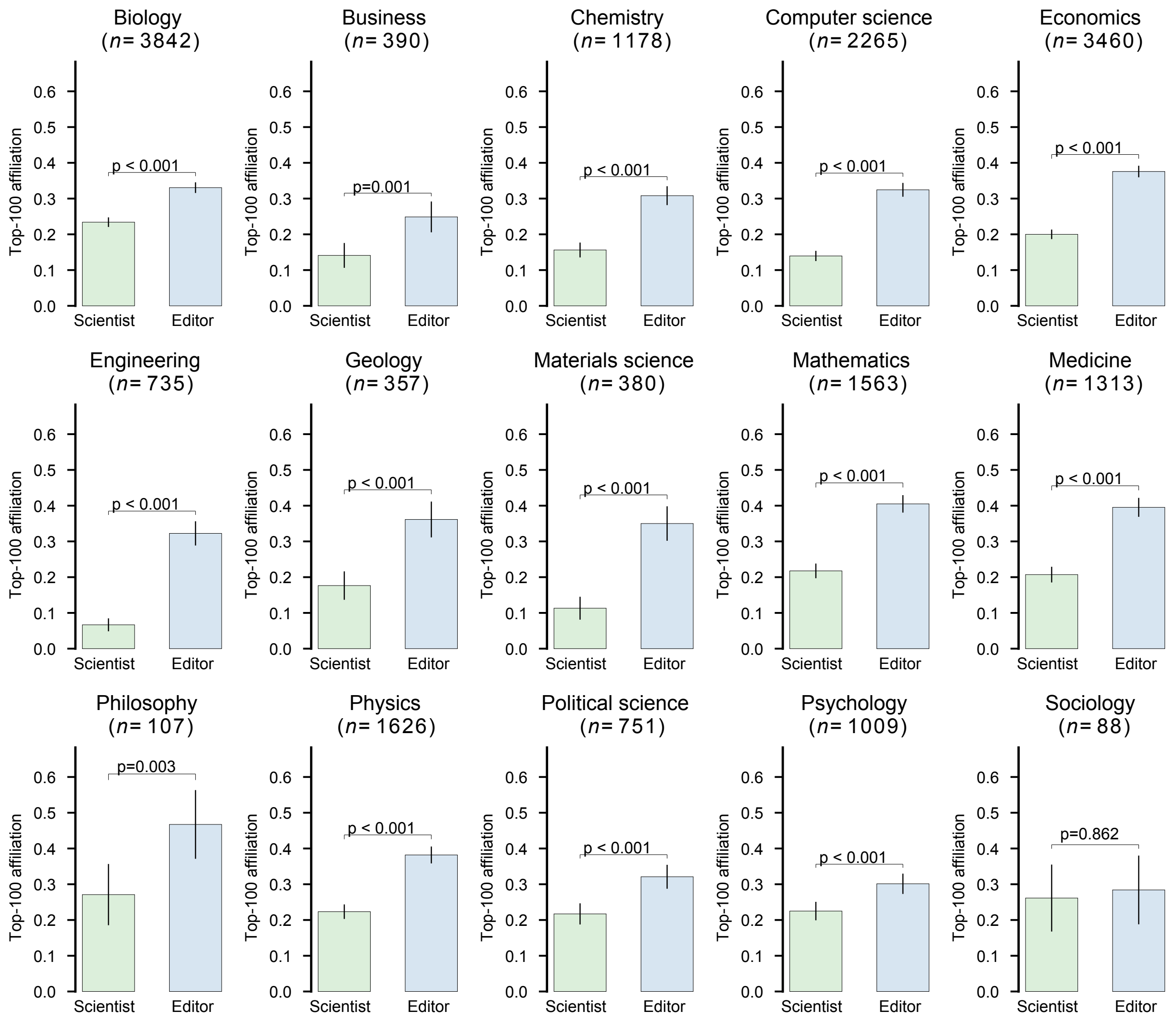

Supplementary Figure 6: **Affiliation rank of editors upon the start of editorship across disciplines.** Each editor is compared to randomly selected scientists whose first year of publication matches that of the editor. The percentage of those whose affiliation ranks among the top 100 is measured at the year that precedes the start of the editorship, with error bars representing the 95% confidence intervals. p-values are calculated using Fisher's exact tests. Unless otherwise specified in figure, all exact p-values are less than 10^{-250}. Sample size in each discipline is denoted in the title of each panel. Data are presented as mean values $+/-$ 95% confidence intervals.

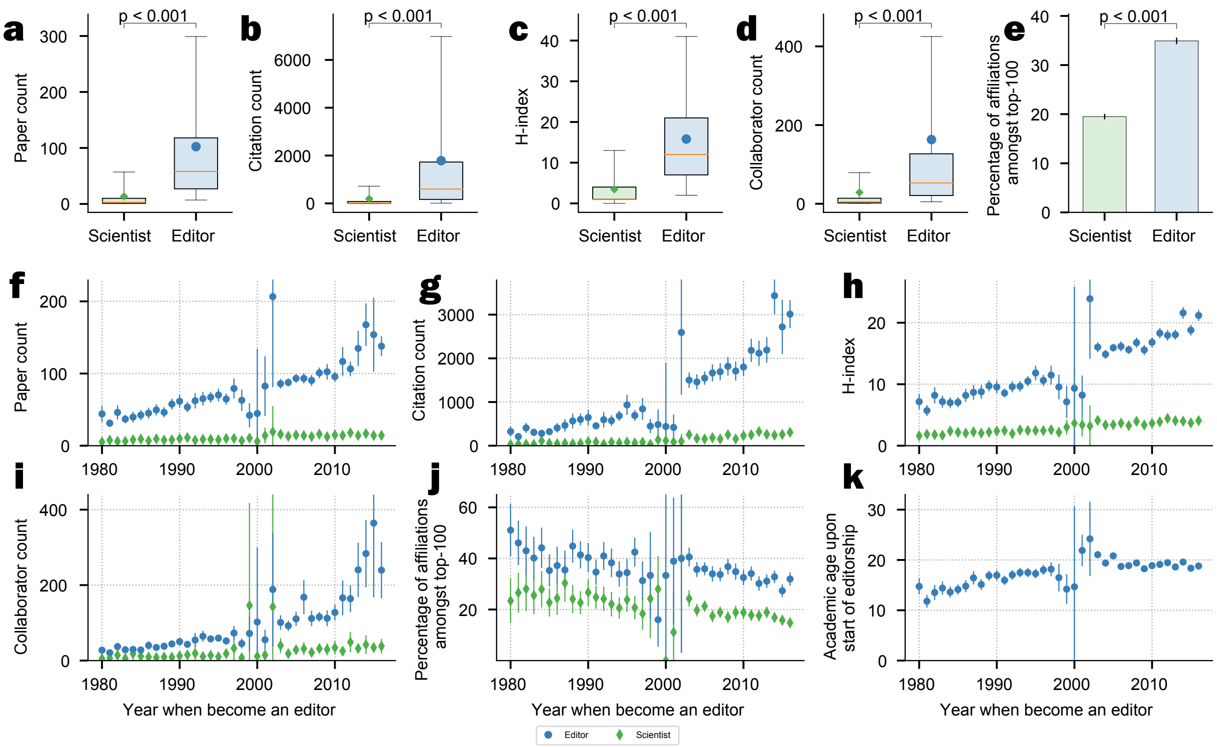

Supplementary Figure 7: **Editors' characteristics upon the start of editorship.** The same analysis as shown in Figure 1 but 50 scientists, instead of a single scientist, are sampled (with replacement) to be compared with each editor. p-values are calculated using two-sided Welch's T-test (**a–d**) and Fisher's exact test (**e**). All exact p-values are less than 10^{-250}. Data are presented as mean values $+/-$ 95% confidence intervals (**e–k**).

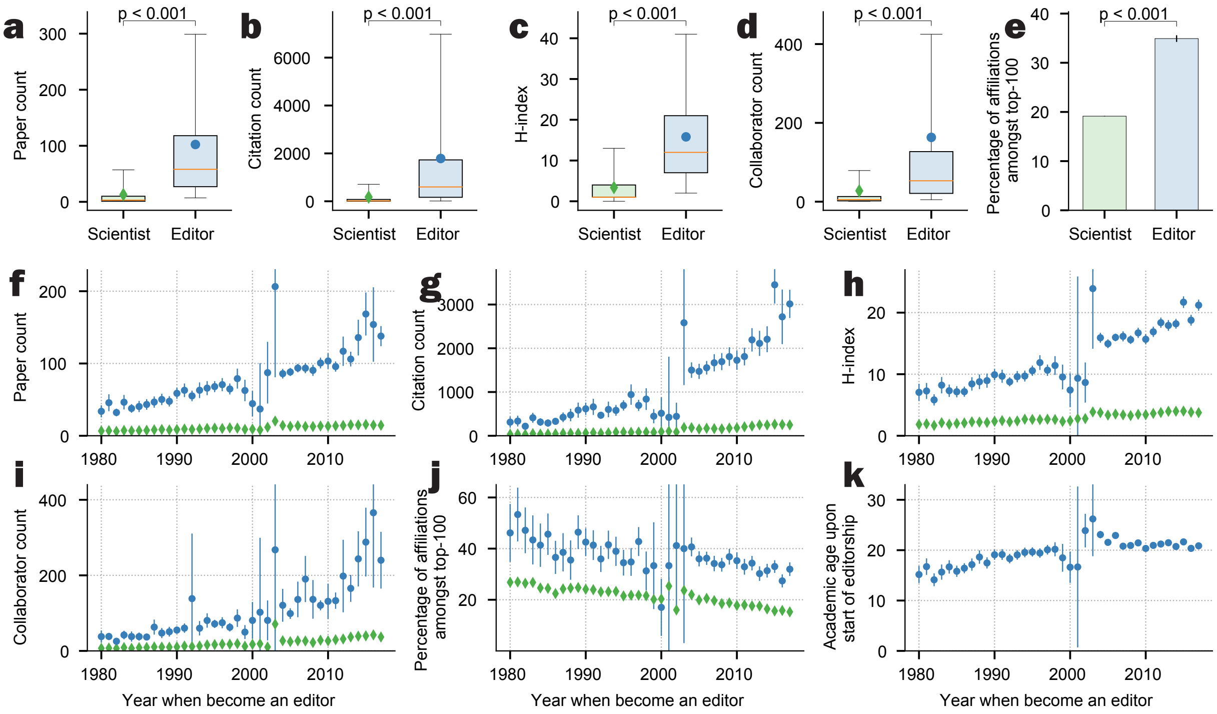

Supplementary Figure 8: **Editors' characteristics upon the start of editorship.** The same analysis as shown in Figure 1 but 200 scientists, instead of a single scientist, are sampled (with replacement) to be compared with each editor. p-values are calculated using two-sided Welch's T-test (**a–d**) and Fisher's exact test (**e**). All exact p-values are less than 10^{-250}. Data are presented as mean values $+/-$ 95% confidence intervals (**e–k**).

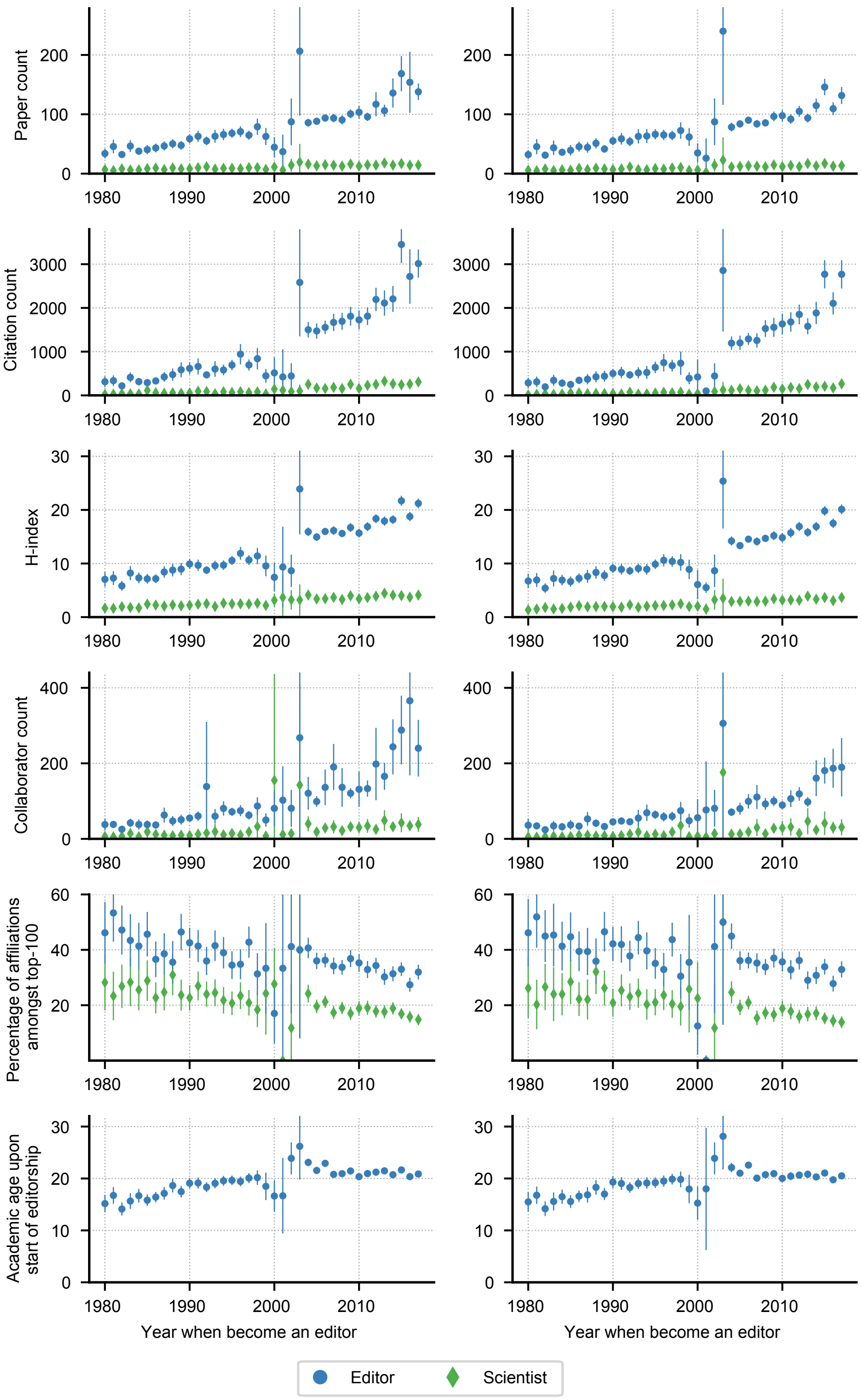

Supplementary Figure 9: **Editors' temporal trends in all disciplines (left column, $n = 19,064$) and in all disciplines except Biology (right column, $n = 15,222$).** Dots represent sample mean, and error bars represent 95% confidence intervals.

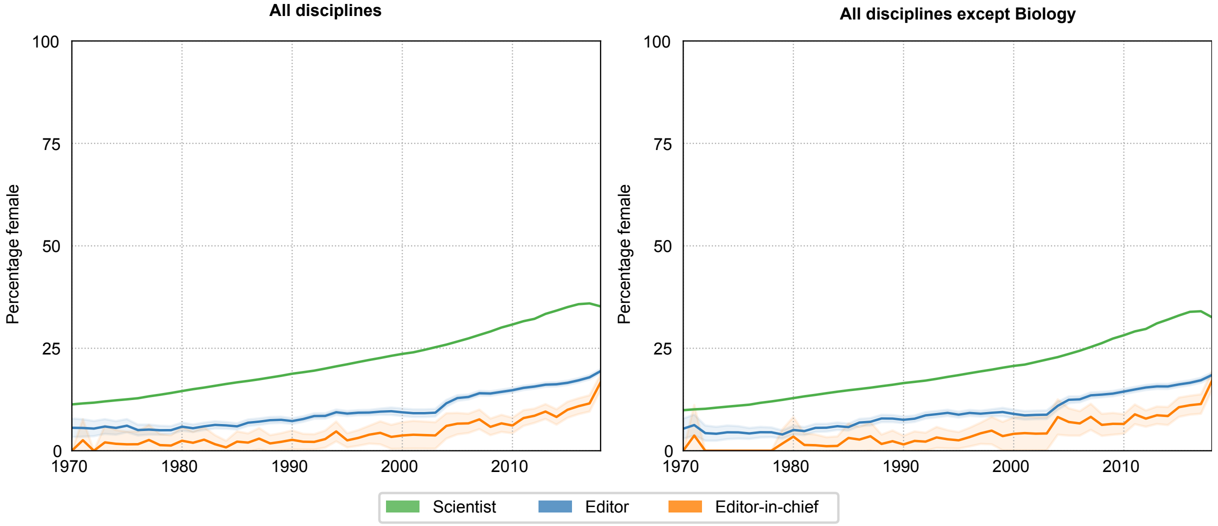

Supplementary Figure 10: **Gender disparity over time in all disciplines (left panel) and in all disciplines except Biology (right panel).** Data are presented as mean values $+/-$ 95% confidence intervals (shaded regions).

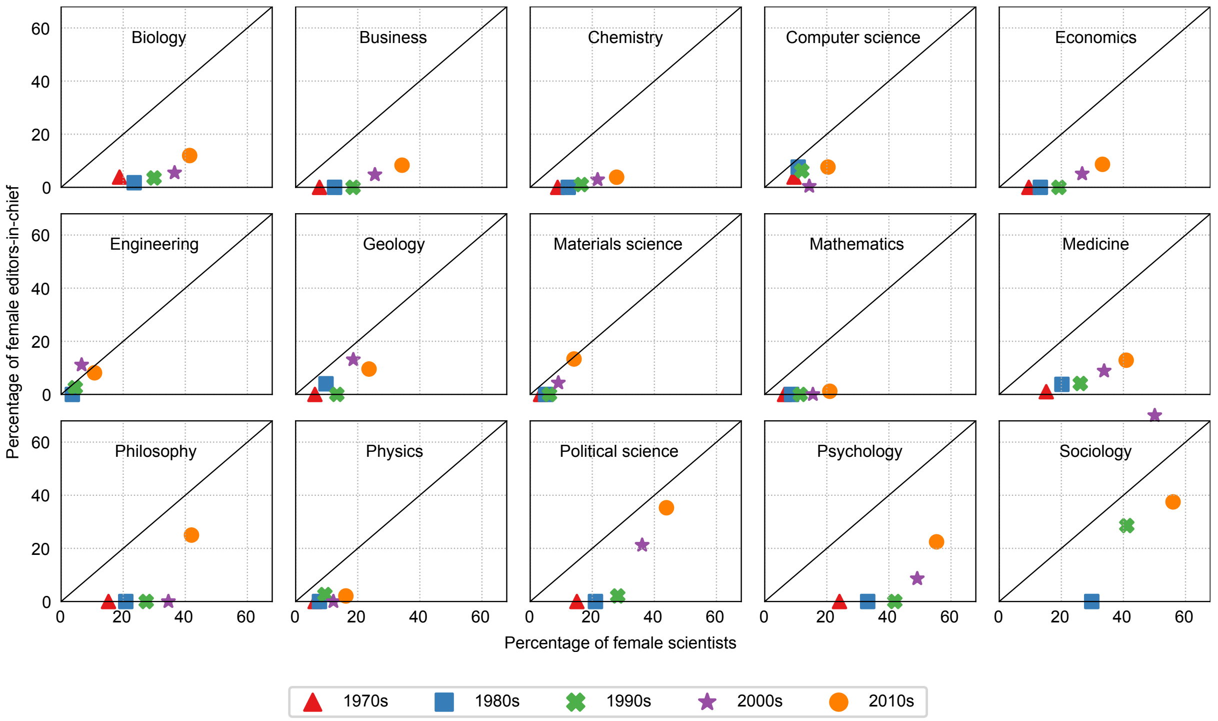

Supplementary Figure 11: **Percentage of female editors-in-chief against that of female scientists across disciplines over the past five decades.** The triangles, squares, crosses, stars, and circles correspond to the 1970s, 1980s, 1990s, 2000s, and 2010s, respectively.

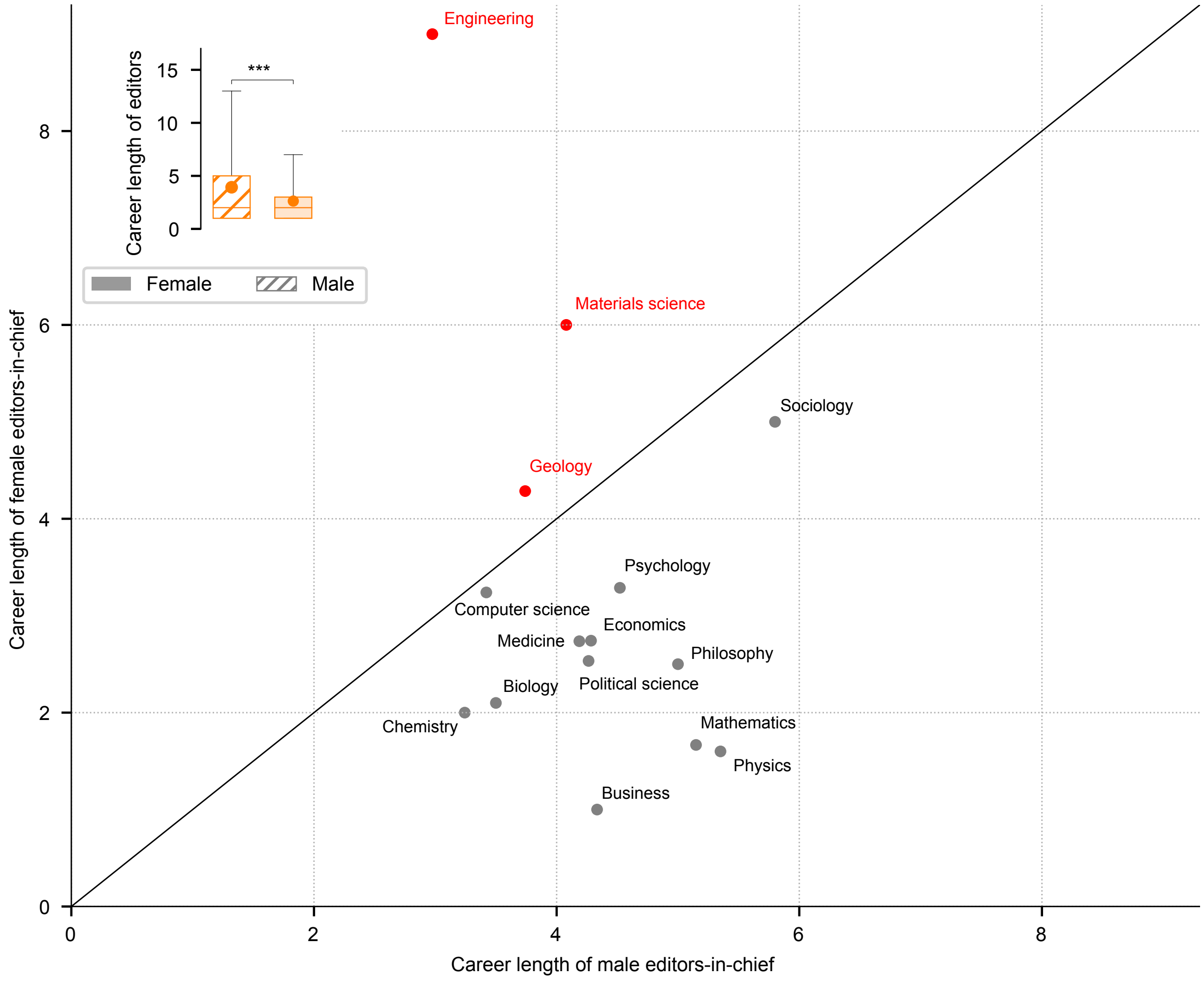

Supplementary Figure 12: **Career length of editors-in-chief.** Career length of female editors-in-chief ($n = 565$) against that of their male counterparts ($n = 4,122$) across disciplines. Red highlights the disciplines in which the career length of women is greater than that of men. The inset shows the average career lengths, taken over all disciplines. Circles and diamonds represent the sample mean of man and woman, respectively; the boxes extend from the lower to upper quartile values of the data, with a line at the median; whiskers extend until the 5-th and the 95-th percentile. p-values are calculated using two-sided Welch's T-test (inset), and *** means $p < 0.001$. The exact p-value is 3.94e-10.

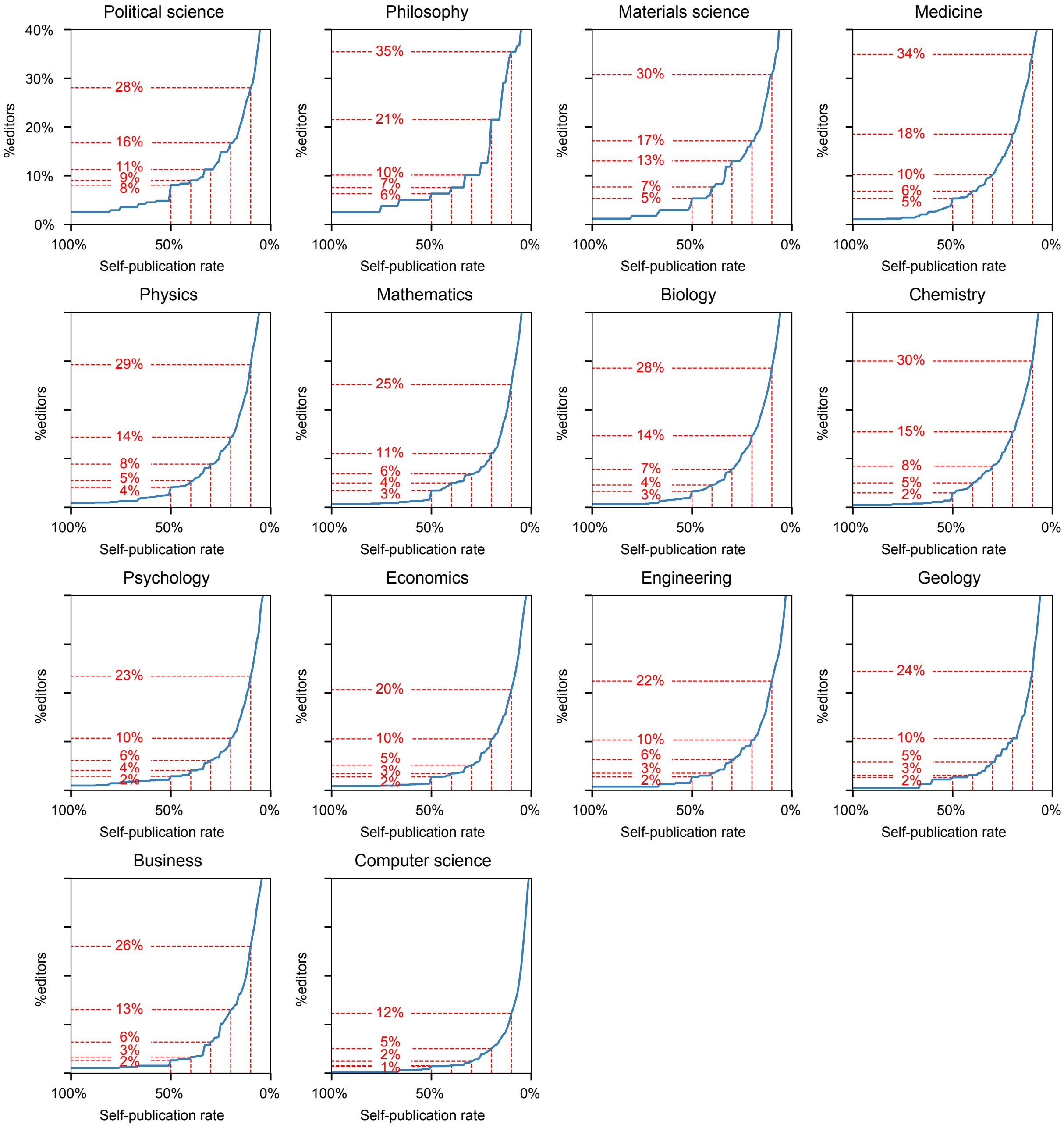

Supplementary Figure 13: **Cumulative distribution of editors' self-publication rate in each discipline, highlighting the proportion of those whose rate is** $\geq 10\%, \geq 20\%, \ldots, \geq 50\%$**.**

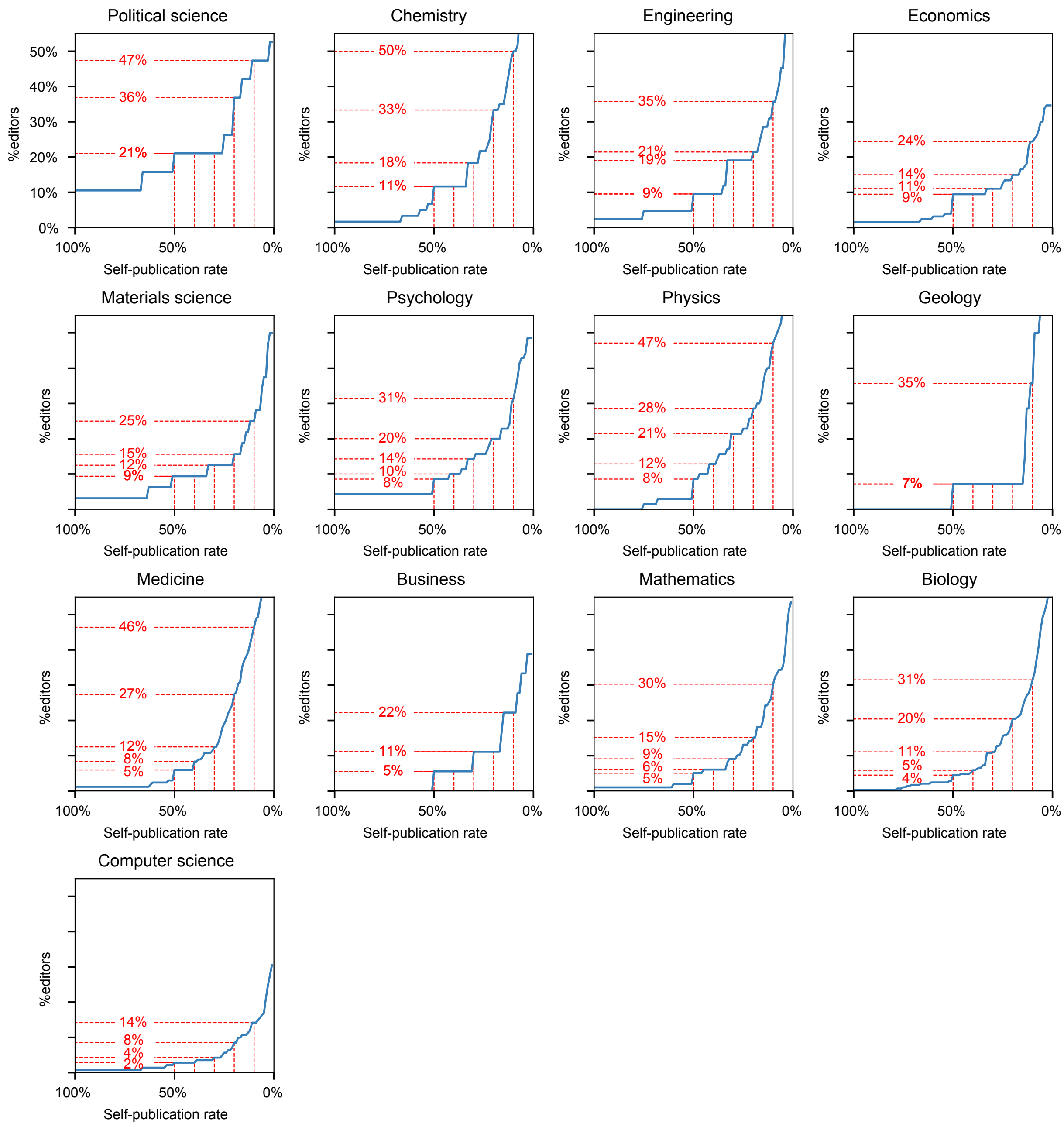

Supplementary Figure 14: **Cumulative distribution of editors-in-chief's self-publication rate in each discipline, highlighting the proportion of those whose rate is $\geq 10\%, \geq 20\%, \ldots, \geq 50\%$.**

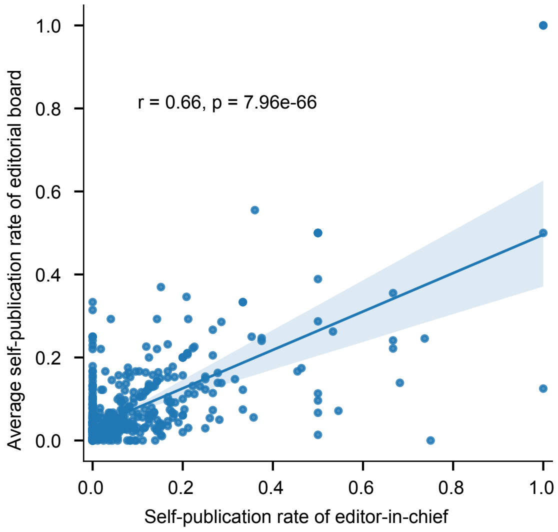

Supplementary Figure 15: **Correlation between the self-publication rates of the editors-in-chief and their editorial boards.** Figure reports two-sided Pearson correlation. Shaded regions represent 95% confidence intervals of the regression estimate.

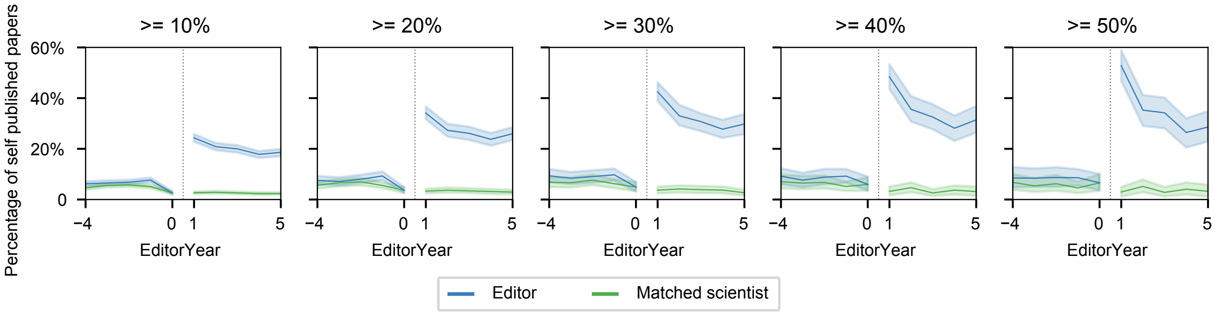

Supplementary Figure 16: **Comparing editors whose self-publication rate is ≥10%, ≥20%, . . ., ≥50% to their matched scientists.** The self-publication rates of (e, j) and their matched scientists are matched up to $\text{year}_0^{(e,j)}$. This figure is similar to the upper row of Figure 3f except that it includes the years before, and not just after, e becomes an editor. Data are presented as mean values $+/-$ 95% confidence intervals.

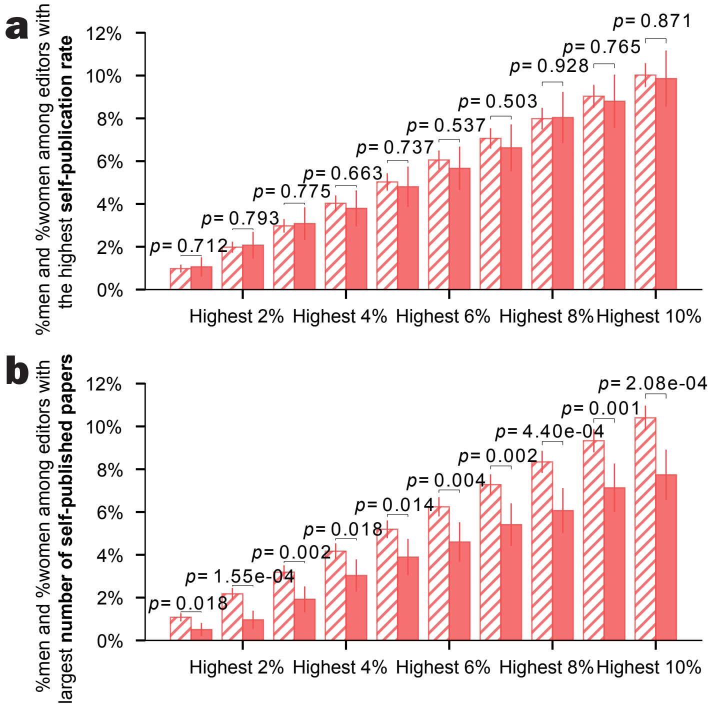

Supplementary Figure 17: **Gender differences in self-publishing behavior.** **a**, Out of all men and women, the percentage of those who fall among the top 1%, 2%, $\dots$, and 10% of editors with the highest self-publication rates; p-values are calculated using two-sided Fisher's exact tests, $n_{\text{male}} = 11,017$, $n_{\text{female}} = 1,978$. **b**, The same as (**a**), but for those who have the largest number of papers published in their own journal.

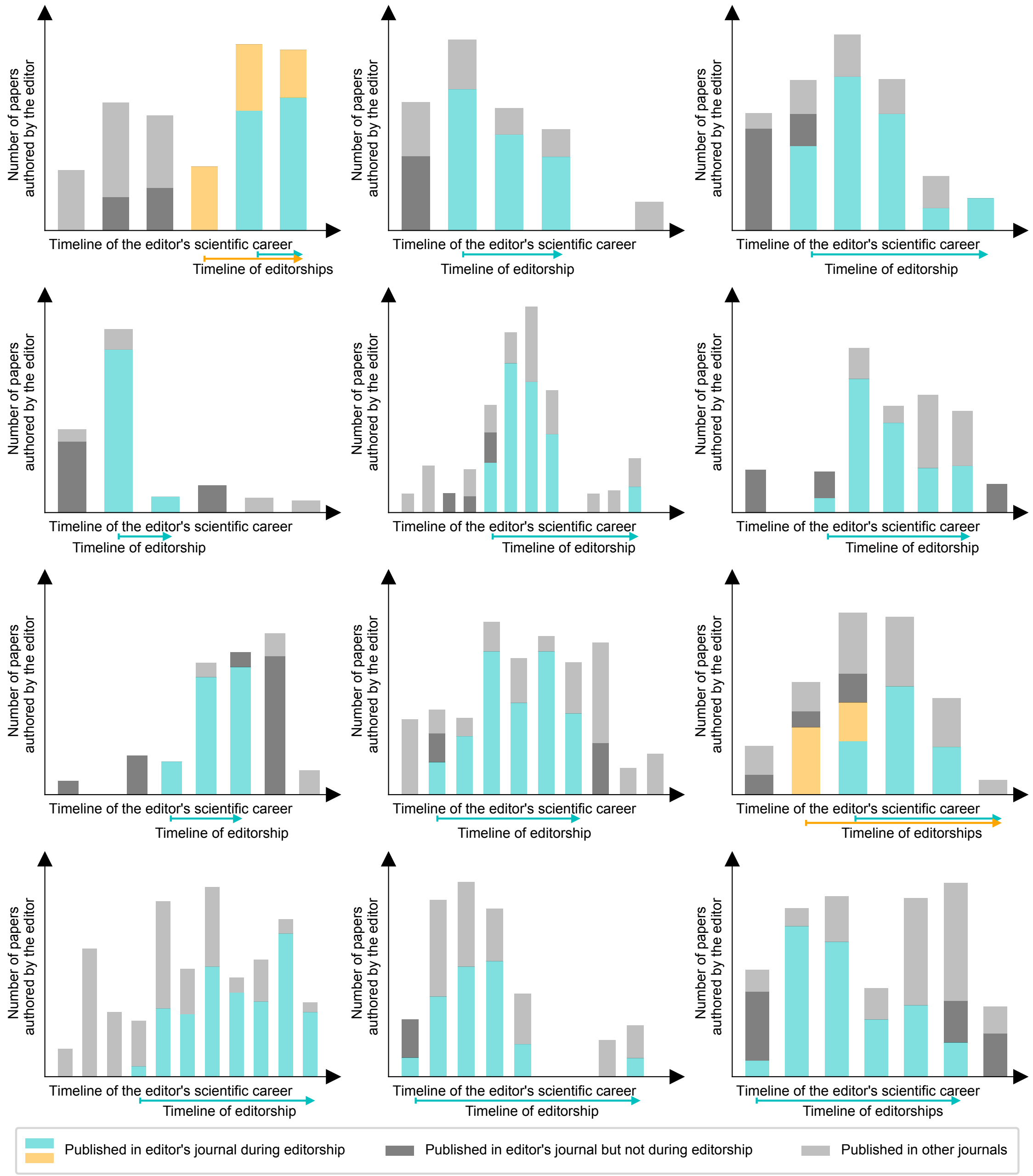

Supplementary Figure 18: **Extreme editors continued.** Out of all editors who publish at least 30 papers throughout their careers, we analyze the 15 who have the highest proportion of their papers published in the journal(s) they are editing. Figures 4**a** to 4**c** in the main manuscript correspond to the top 3, while this figure corresponds to the remaining 12. To preserve anonymity, ticks on the x-axis and y-axis are hidden and Gaussian noise is added to the bar heights. For each of these editors, we show the total number of papers they publish as well as how many of those papers are published in the editor's journal(s); results are aggregated over five-year periods to preserve anonymity. The horizontal line(s) underneath the plot represent the span of the editorship(s).

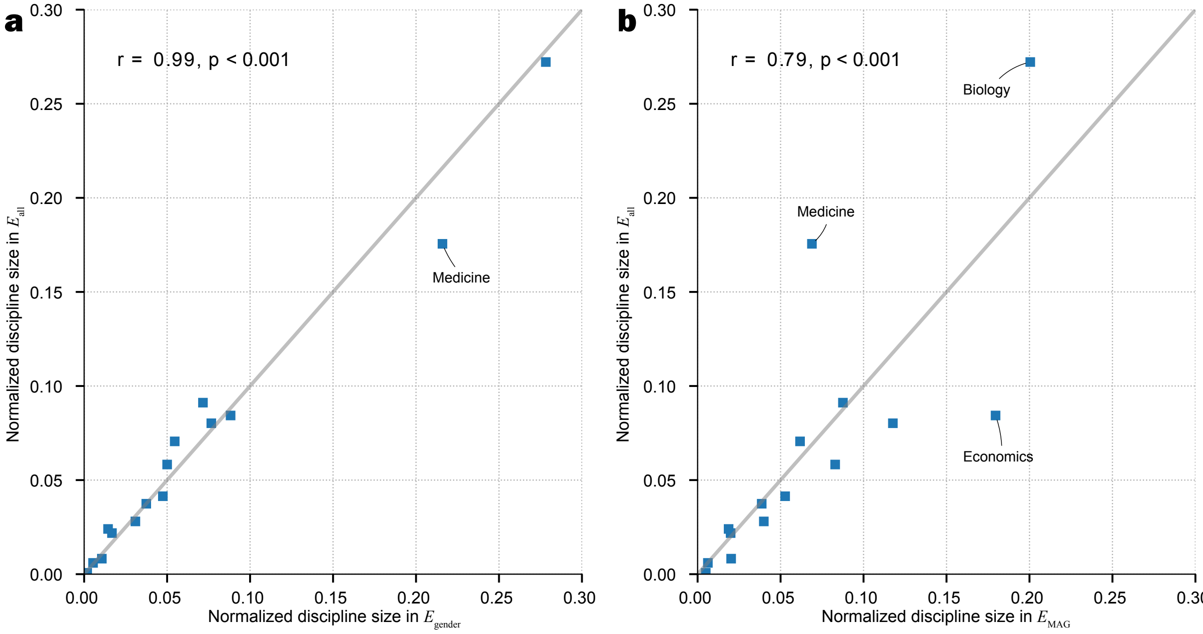

Supplementary Figure 19: **The correlation (a) between the normalized discipline sizes in E_{gender} and E_{all}, and (b) between the normalized discipline sizes in E_{MAG} and E_{all}.** r and p represent the two- sided Pearson correlation coefficient and the corresponding p-value. In (**a**), the exact p-value is 7.46e-12. In (**b**), the exact p-value is 5.18e-4.

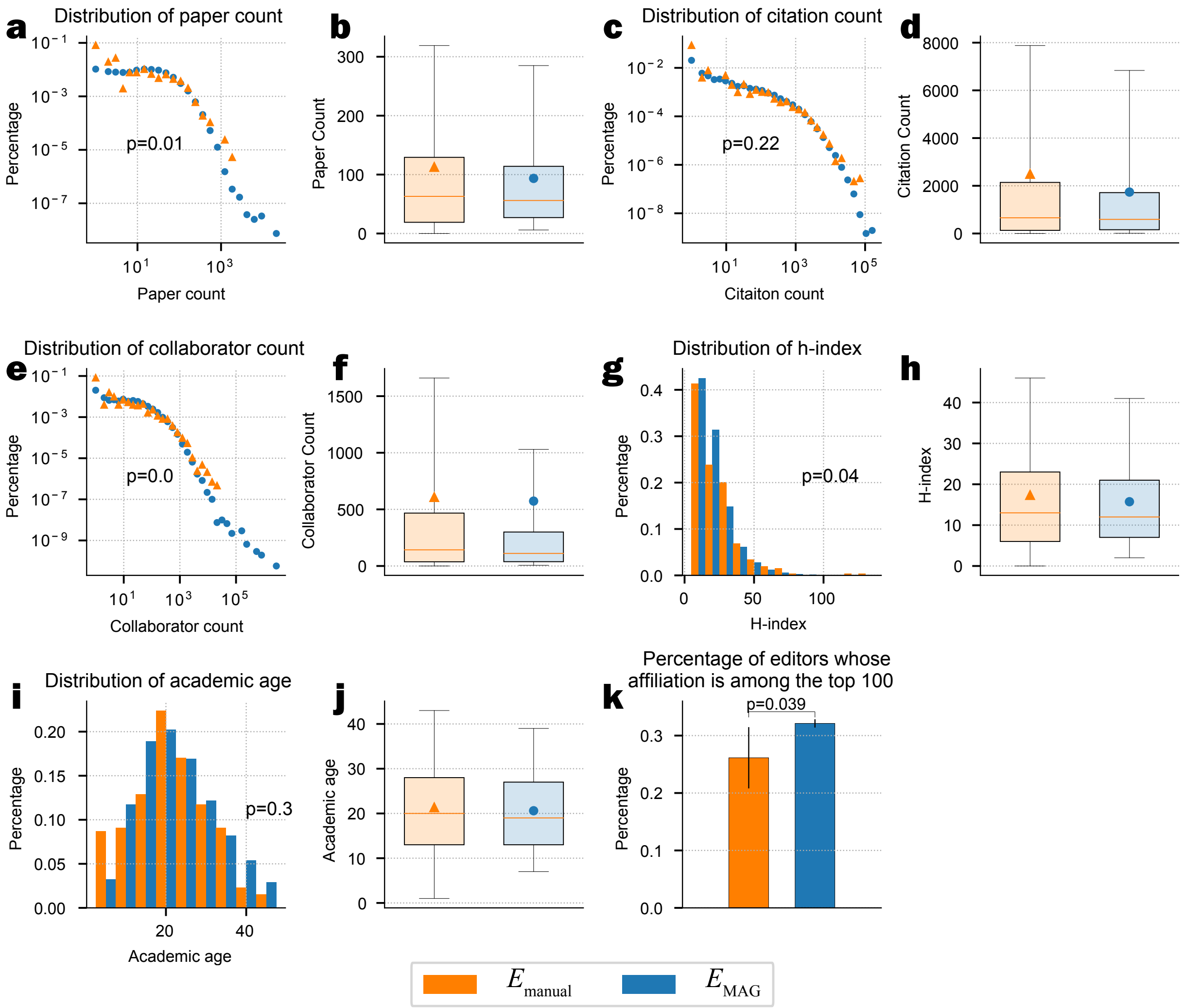

Supplementary Figure 20: **Comparing manually-identified editors** (E_{manual}, $n = 264$) **to those whose MAG entries were identified** (E_{MAG}, $n = 19,741$)**.** The two sets of editors are compared in terms of paper count (**a**)-(**b**), citation count (**c**)-(**d**), collaborator count (**e**)-(**f**), h-index (**g**)-(**h**), academic age (**i**)-(**j**), and percentage of those affiliated with top-100 institutions (**k**); descriptive statistics are measured at the year preceding the start of the editorship. A t-test is used to test statistical significance in (**k**), while Kolmogorov-Smirnov tests are used in (**a**), (**c**), (**e**), (**g**), and (**i**); the corresponding p-values displayed in each subfigure. Circles and diamonds in the box plots represent the sample mean of E_{MAG} and E_{manual}, respectively; the boxes extend from the lower to upper quartile values of the data, with a line at the median; whiskers extend until the 5-th and the 95-th percentile. Data in (**k**) are presented as mean values $+/-$ 95% confidence intervals. p-values are calculated using two-tailed Student's t-tests.

Supplementary Tables

Supplementary Table 1: **Comparing our study (red text) to other papers studying editors' self-publishing.**

Reference	Publication year	Discipline(s) analyzed	No. journals analyzed	Year(s) analyzed
Liu et al. (this study)	2022	Biology, Business, Chemistry, Computer Science, Economics, Engineering, Geography, Geology, Material Science, Mathematics, Medicine, Philosophy, Physics, Political Science, Psychology, Sociology	1,709	1980–2018
Campanario [1]	1996	Educational Psychology	18	1987-1988
Luty et al. [2]	2009	Medicine	20	2006
Bošnjak et al. [3]	2011	Natural Science / Engineering, Medicine, Humanities, and Social Sciences (Croatian journals)	180	2005-2008
Mani et al. [4]	2013	Urology	5	2006-2010
Medoff [5]	2013	Economics	6	1990
Rösing et al. [6]	2014	Oral Health	10	2010-2012
Walters [7]	2015	Library & Information Science	30	2007-2012
Zdeněk & Lososová [8]	2018	Economics	17	2012-2016
Zdeněk [9]	2018	Economics (Czech journals)	12	2012-2015
Youk and Park [10]	2019	Communications	17	2007-2016
Sen-Crowe et al. [11]	2020	Surgery	10	2016-2019
Xu et al. [12]	2021	Library & Information Science	2	2003-2019

Supplementary Table 2: **Comparing our study (red text) to other papers studying the gender gap in scientific editorship.**

Reference	Publication year	Discipline(s) analyzed	No. journals analyzed	Year(s) analyzed
Liu et al. (this study)	2022	Biology, Business, Chemistry, Computer Science, Economics, Engineering, Geology, Material Science, Mathematics, Medicine, Philosophy, Physics, Political Science, Psychology, Sociology	1,709	1970–2018
Teghtsoonian [13]	1974	Psychology	11	1970–1972
White [14]	1985	Psychology	14	1972, 1977, 1982
Pion et al. [15]	1996	Psychology	5	1971, 1981, 1991
Dickersin et al. [16]	1998	Epidemiology	4	1982, 1987, 1992, 1994
Robinson et al. [17]	1998	Educational Psychology	6	1976–1996
McSweeney et al. [18]	2000	Applied Behavior Analysis	5	1978–1997
Kennedy et al. [19]	2001	Medicine	12	1999
Addis and Villa [20]	2003	Economics	36	1970–1996
Keiser [21]	2003	Medicine	6	1993, 2003
Evans et al. [22]	2005	Psychology	6	2004
Morton & Sonnad [23]	2007	Medicine	54	2004
Jagsi et al. [24]	2008	Medicine	5	1970, 1975, 1980, 1990, 1995, 2000, 2005
Fong et al. [25]	2009	Psychology	20	2003–2008
Metz and Harzing [26]	2009	Management	57	1989, 1994, 1999, 2004
Amrein et al. [27]	2011	Medicine	60	2011
Stegmaier et al. [28]	2011	Political Science	50	2010
Choi and Miller [29]	2012	Otolaryngology	6	2010
Metz and Harzing [30]	2012	Management	57	1989–2009
Okike et al. [31]	2012	Orthopedics	2	1970, 1980, 1990, 2000, 2007
Mauleón et al. [32]	2013	All disciplines (Spanish journals)	131	1998-2009
Cho et al. [33]	2014	Environmental biology, natural resource management, and plant sciences	10	1985–2013
Erren et al. [34]	2014	Medicine	6	2010, 2011
Ioannidou et al. [35]	2015	Dentistry	69	2014

Metz et al. [36]	2016	Management	52	1989, 1994, 1999, 2004, 2009
Topaz and Sen [37]	2016	Mathematical Sciences	435	2016
Dhanani and Jones [38]	2017	Accounting	50	1999–2009
Gollins et al. [39]	2017	Dermatology	25	1868–2017
Piper et al. [40]	2018	Radiology	4	1973–2017
Fox et al. [41]	2019	Ecology and Evolution	6	2003–2015
Hafeez et al. [42]	2019	Psychiatry	119	2018
Harris et al. [43]	2019	Surgery	10	1997, 2007, 2017
Jalilianhasanpour et al. [44]	2019	Radiology	9	2002–2017
Kaji et al. [45]	2019	Emergency Medicine	1	2018
Litvack et al. [46]	2019	Otolaryngology	9	1997–2017
Lorello et al. [47]	2019	Anesthesia	1	1954–2018
Pagel et al. [48]	2019	Cardiothoracic and Vascular Anesthesia	1	1987–2019
Alonso-Arroyo et al. [49]	2020	Pediatrics	125	2020
Balasubramanian et al. [50]	2020	Cardiology	22	1998, 2003, 2008, 2013, 2018
Rynecki et al. [51]	2020	Orthopedics	4	1997, 2007, 2017
Sarna et al. [52]	2020	Medicine, Nursing, and Pharmacy	21	1995–2016
Schurr et al. [53]	2020	Geography	22	1999, 2017
Alkhawtani et al. [54]	2021	Radiology	57	2020
Bennie and Koka [55]	2021	Prosthodontics	28	2020
Gottlieb et al. [56]	2021	Emergency Medicine	37	2019
Hutchinson et al. [57]	2021	Emergency Medicine	17	2019
Pflibsen et al. [58]	2021	Plastic Surgery	3	2009–2018
Pinho-Gomes et al [59]	2021	Medicine	410	2019
Salazar et al. [60]	2021	Medicine	25	2021
Sperotto et al. [61]	2021	Biotechnology	50	2021
Palser et al. [62]	2022	Psychology & Neuroscience	100	2020

Supplementary Table 3: **The number of collected editors ($n = 102,964$) in each discipline.**

Discipline	Number of editors	Percentage
Biology	27914	27.11
Medicine	18005	17.49
Physics	9348	9.08
Economics	8649	8.40
Computer science	8226	7.99
Chemistry	7238	7.03
Mathematics	5977	5.80
Psychology	4248	4.13
Engineering	3839	3.73
Political science	2875	2.79
Geology	2464	2.39
Materials science	2240	2.18
Business	1155	1.12
Philosophy	614	0.60
Sociology	172	0.17

Supplementary Table 4: **The number of editors whose gender is classified with a confidence of 90% or above ($n = 80,776$) in each discipline.**

Discipline	Number of editors	Percentage
Biology	22474	27.82
Medicine	17442	21.59
Economics	7129	8.83
Computer science	6190	7.66
Physics	5783	7.16
Chemistry	4409	5.46
Mathematics	4040	5.00
Psychology	3831	4.74
Engineering	3024	3.74
Political science	2494	3.09
Materials science	1354	1.68
Geology	1164	1.44
Business	861	1.07
Philosophy	433	0.54
Sociology	148	0.18

Supplementary Table 5: **The number of editors matched to scientists in MAG** ($n = 19,698$) **in each discipline.**

Discipline	Number of editors	Percentage
Biology	3949	20.05
Economics	3537	17.96
Computer science	2318	11.77
Physics	1725	8.76
Mathematics	1633	8.29
Medicine	1357	6.89
Chemistry	1216	6.17
Psychology	1039	5.27
Political science	786	3.99
Engineering	761	3.86
Business	398	2.02
Materials science	392	1.99
Geology	368	1.87
Philosophy	122	0.62
Sociology	97	0.49

Supplementary Table 6: **Scientists matched to editors in Figure 3f have similar self-publication rates up to and including year$_0^{(e,j)}$.**

Self-publication rate cohort	Year since becoming editor	Author mean	Editor mean	Standardized difference	P-value (Wilcoxon test)	P-value (K-S test)
≥ 0.5	-4	0.067	0.085	0.077	0.500	1.000
	-3	0.054	0.084	0.140	0.132	0.969
	-2	0.062	0.087	0.104	0.307	1.000
	-1	0.046	0.086	0.208	0.038	0.561
	0	0.066	0.066	0.002	0.977	1.000
≥ 0.4	-4	0.070	0.092	0.098	0.348	1.000
	-3	0.065	0.076	0.054	0.443	0.999
	-2	0.068	0.088	0.090	0.271	0.999
	-1	0.052	0.092	0.203	0.019	0.464
	0	0.059	0.060	0.003	0.989	1.000
≥ 0.3	-4	0.068	0.094	0.115	0.106	0.911
	-3	0.066	0.084	0.081	0.274	0.781
	-2	0.077	0.090	0.061	0.411	0.998
	-1	0.062	0.098	0.168	0.023	0.520
	0	0.049	0.050	0.002	0.990	1.000
≥ 0.2	-4	0.056	0.075	0.100	0.065	0.770
	-3	0.066	0.071	0.024	0.384	0.833
	-2	0.070	0.080	0.048	0.563	0.959
	-1	0.055	0.093	0.190	0.002	0.134
	0	0.036	0.037	0.001	0.998	1.000
≥ 0.1	-4	0.046	0.062	0.094	0.018	0.310
	-3	0.056	0.065	0.051	0.090	0.304
	-2	0.058	0.068	0.059	0.038	0.236
	-1	0.051	0.076	0.145	$p < 0.001$	0.025
	0	0.026	0.026	0.001	0.993	1.000

Supplementary Table 7: **Regression-estimated temporal trend of the number of papers e publishes in j during the 5 years before, and the 5 years after, e becomes an editor of j.** The regression model controls for gender and journal fixed effects. More specifically, it is specified as follows: $Y_{it} = \beta_0 + \beta_1 * D_{it} + \beta_2 * (t - T_i) + \beta_3 * (t - T_i) * D_{it} + \beta_4 * G_i + \beta_5 * G_i * D_{it} + \beta_j + \epsilon_{it}$. In the model, the subscript i denotes an editor-journal pair, (e, j), while t denotes the year when an observation on (e, j) is made. Y_{it} is the number of papers e publishes in j in year t (standardized). T_i is $\text{year}_0^{(e,j)}$, implying that $t - T_i$ is the number of years between the year of observation and the year when e starts editing j. D_{it} is a binary indicator of whether t is greater than T_i, and G_i is a binary indicator of whether i is male. β_j is the journal fixed-effect control. p-values are calculated using the Student's t-test, with standard errors clustered at the editor level. The exact p-values of those less than 0.001 are 2.75e-43, 2.921876e-18, and 5.05e-04, respectively.

	b	95% CI	p
After editorship starts (β_1)	0.053	(0.021, 0.086)	0.001
Year since editorship starts (β_2)	0.029	(0.025, 0.034)	< 0.001
After editorship starts × Year since editorship starts (β_3)	-0.031	(-0.038, -0.024)	< 0.001
Male (β_4)	0.046	(0.02, 0.071)	< 0.001
After editorship starts × Male (β_5)	0.035	(0.004, 0.067)	0.027
Observations	119,553		
Adjusted R^2	0.160		

Supplementary Table 8: **Regression-estimated temporal trend of the self-publication rate of e during the 5 years before, and the 5 years after, e becomes an editor of j.** Model is specified in the same way as Supplementary Table 7. The exact p-values of those less than 0.001 are 6.28e-11 and 5.17e-12, respectively.

	b	95% CI	p
After editorship starts (β_1)	0.008	(0.002, 0.014)	0.008
Year since editorship starts (β_2)	0.003	(0.002, 0.003)	< 0.001
After editorship starts $\times$ Year since editorship starts (β_3)	-0.004	(-0.006, -0.003)	< 0.001
Male (β_4)	-0.005	(-0.01, -0.001)	0.025
After editorship starts $\times$ Male (β_5)	0.007	(0.001, 0.012)	0.017
Observations	119553		
Adjusted R^2	0.087		

Supplementary Note 1: Inferring the Discipline of Authors and Journals

The papers in MAG are categorized into 19 high-level disciplines, which are further divided into more than 10,000 lower-level disciplines. A high-level discipline may have multiple lower-level "children", and each such child may have multiple high-level "parents". Let us denote a high-level discipline by D and a lower-level one by d. Moreover, if the former is a parent of the latter, we write $d \in D$. In MAG, every paper, p, is associated with a discipline, d, with a confidence score denoted by $score(p, d) \in [0, 1]$. Based on this information, we need to compute a score representing our confidence that p belongs to a given high-level discipline, D. To this end, we sum up $score(p, d)$ for every $d \in D$, as long as $score(p, d) \geq 0.5$. More formally:

$$score(p, D) = \sum_{d \in D} score(p, d) \cdot \mathbb{1}_{score(p,d) \geq 0.5}$$

On the other hand, the score indicating our confidence that a scientist s belongs to D is calculated as follows, where P_s denotes the set of papers published by s:

$$score(s, D) = \sum_{p \in P_s} score(p, D)$$

Based on this, the primary discipline of a scientist, s, is computed as follows:

$$\begin{aligned}\mathcal{D}(s) &= \arg\max_D score(s, D) \\ &= \arg\max_D \sum_{p \in P_s} \sum_{d \in D} score(p, d) \cdot \mathbb{1}_{score(p,d) \geq 0.5}\end{aligned}$$

Similarly, the primary discipline of a journal, j, is computed as follows:

$$\begin{aligned}\mathcal{D}(j) &= \arg\max_D score(j, D) \\ &= \arg\max_D \sum_{p \in P_j} \sum_{d \in D} score(p, d) \cdot \mathbb{1}_{score(p,d) \geq 0.5}\end{aligned}$$

where P_j is the set of papers published in j.

Supplementary Note 2: Author Name Disambiguation

In this note, we start by explaining how the Microsoft Academic Graph (MAG) dataset handles both the name conflation problem and the name disambiguation problem, while the second section discusses how this paper deals with name disambiguation when it comes to editors.

0.1 MAG's Approach

When it comes to author entity linking, MAG identifies two main challenges [63, 64]. The first is the *name conflation problem*, which arises from the possibility that an author might be misrepresented as multiple ones. The second is the *name disambiguation problem*, which arises from the possibility that multiple authors might be misrepresented as one. To tackle these two problems, MAG compares the following two sources of information to conflate and disambiguate the authors' names, resulting in 240 million authors.

1. Information about the authors' affiliations, their publication venues, and their collaborators. All of this information is aggregated using machine learning techniques to increase the confidence that two entities represent the same individual.

2. Data mined from the authors' websites and CVs, taking advantage of Microsoft's web-scale infrastructure. More specifically, this infrastructure facilitates the analysis of billions of documents online, all of which are used to train machine learning algorithms to automatically identify pages that may be relevant to the authors. Such pages may contain publication lists, which are then cross-referenced with the Microsoft Academic Knowledge Graph to detect entries that belong to the same author.

Note that the way in which papers are mapped to authors in MAG is rather conservative, as it favors underconflation rather than overconflation. More specifically, any two author entities are not merged unless the probability of them being the same person is extremely high.

Consequently, the MAG team states that they *"have confidence that when Microsoft Academic attributes a set of papers to an author, they were actually written by that person"* [63].

0.2 Identifying editors in MAG

For each Elsevier journal, we identify its editors in MAG using the Editorial Board Information page published in each issue of that journal. This page specifies the name and affiliation of those who edit the issue. Additionally, since we know when an issue is published, we also know when such editors were affiliated with their respective affiliations. Using this information, we identify the publication records of each editor of any given issue by finding the scientist in MAG whose name matches that of the editor, and who has the same affiliation as the editor in the year when that issue was published. To avoid ambiguity, we only consider editors who are uniquely identified in MAG.

Supplementary References

[1] Campanario, J. M. The competition for journal space among referees, editors, and other authors and its influence on journals' impact factors. *J. Am. Soc. Inf. Sci.* **47**, 184–192 (1996).

[2] Luty, J., Arokiadass, S., Easow, J. & Anapreddy, J. Preferential publication of editorial board members in medical specialty journals. *J. Med. Ethics* **35**, 200–202 (2009).

[3] Bošnjak, L., Puljak, L., Vukojević, K. & Marušić, A. Analysis of a number and type of publications that editors publish in their own journals: case study of scholarly journals in Croatia. *Scientometrics* **86**, 227–233 (2011).

[4] Mani, J. *et al.* I publish in I edit? Do editorial board members of urologic journals preferentially publish their own scientific work? *PLOS One* **8**, e83709 (2013).

[5] Medoff, M. H. Editorial favoritism in economics? *South. Econ. J.* 425–434 (2003).

[6] Rösing, C. K., Junges, R. & Haas, A. N. Publication rates of editorial board members in oral health journals. *Braz. Oral. Res.* **28**, 1–5 (2014).

[7] Walters, W. H. Do editorial board members in library and information science publish disproportionately in the journals for which they serve as board members? *J. Sch. Publ.* **46**, 343–354 (2015).

[8] Zdeněk, R. & Lososová, J. An analysis of editorial board members' publication output in agricultural economics and policy journals. *Scientometrics* **117**, 563–578 (2018).

[9] Zdeněk, R. Editorial board self-publishing rates in czech economic journals. *Sci. Eng. Ethics* **24**, 669–682 (2018).

[10] Youk, S. & Park, H. S. Where and what do they publish? Editors' and editorial board members' affiliated institutions and the citation counts of their endogenous publications in the field of communication. *Scientometrics* **120**, 1237–1260 (2019).

[11] Sen-Crowe, B. *et al.* Variations in surgical peer-reviewed publications among editorial board members, associate editors and their respective journal: towards maintaining academic integrity. *Ann. Med. Surg.* **60**, 140–145 (2020).

[12] Xu, S., An, M. & An, X. Do scientific publications by editorial board members have shorter publication delays and then higher influence? *Scientometrics* 1–17 (2021).

[13] Teghtsoonian, M. Distribution by sex of authors and editors of psychological journals, 1970-1972: are there enough women editors? *Am. Psychol.* **29**, 262 (1974).

[14] White, A. Women as authors and editors of psychological journals: a 10-year perspective. *Am. Psychol.* **40**, 527 (1985).

[15] Pion, G. M. *et al.* The shifting gender composition of psychology: trends and implications for the discipline. *Am. Psychol.* **51**, 509 (1996).

[16] Dickersin, K., Fredman, L., Flegal, K. M., Scott, J. D. & Crawley, B. Is there a sex bias in choosing editors? Epidemiology journals as an example. *JAMA* **280**, 260–264 (1998).

[17] Robinson, D. H., McKay, D. W., Katayama, A. D. & Fan, A.-C. Are women underrepresented as authors and editors of educational psychology journals? *Contemp. Educ. Psychol.* **23**, 331–343 (1998).

[18] McSweeney, F. K., Donahoe, P. & Swindell, S. Women in applied behavior analysis. *Behav. Anal.* **23**, 267–277 (2000).

[19] Kennedy, B. L., Lin, Y. & Dickstein, L. J. Women on the editorial boards of major journals. *Acad. Med.* **76**, 849–851 (2001).

[20] Addis, E. & Villa, P. The editorial boards of Italian economics journals: women, gender, and social networking. *Fem. Econ.* **9**, 75–91 (2003).

[21] Keiser, J., Utzinger, J. & Singer, B. H. Gender composition of editorial boards of general medical journals. *Lancet* **362**, 1336 (2003).

[22] Evans, J., Hsieh, P. P.-H. & Robinson, D. H. Women's involvement in educational psychology journals from 1976 to 2004. *Educ. Psychol. Rev* **17**, 263–271 (2005).

[23] Morton, M. J. & Sonnad, S. S. Women on professional society and journal editorial boards. *J. Natl. Med. Assoc.* **99**, 764 (2007).

[24] Jagsi, R., Tarbell, N. J., Henault, L. E., Chang, Y. & Hylek, E. M. The representation of women on the editorial boards of major medical journals: a 35-year perspective. *Archives of internal medicine* **168**, 544–548 (2008).

[25] Fong, C. J., Yoo, J. H., Jones, S. J., Torres, L. G. & Decker, M. L. Trends in female authorships, editorial board memberships, and editorships in educational psychology journals from 2003 to 2008. *Educ. Psychol. Rev* **21**, 267–277 (2009).

[26] Metz, I. & Harzing, A.-W. Gender diversity in editorial boards of management journals. *Acad. Manag. Learn. Educ.* **8**, 540–557 (2009).

[27] Amrein, K., Langmann, A., Fahrleitner-Pammer, A., Pieber, T. R. & Zollner-Schwetz, I. Women underrepresented on editorial boards of 60 major medical journals. *Gend. Med.* **8**, 378–387 (2011).

[28] Stegmaier, M., Palmer, B. & Van Assendelft, L. Getting on the board: the presence of women in political science journal editorial positions. *PS - Political Sci. Politics* **44**, 799–804 (2011).

[29] Choi, S. S. & Miller, R. H. Women otolaryngologist representation in specialty society membership and leadership positions. *Laryngoscope* **122**, 2428–2433 (2012).

[30] Metz, I. & Harzing, A.-W. An update of gender diversity in editorial boards: a longitudinal study of management journals. *Pers.* (2012).

[31] Okike, K. *et al.* The orthopedic gender gap: Trends in authorship and editorial board representation over the past 4 decades. *Am. J. Orthop. (Belle Mead NJ)* **41**, 304–310 (2012).

[32] Mauleón, E., Hillán, L., Moreno, L., Gómez, I. & Bordons, M. Assessing gender balance among journal authors and editorial board members. *Scientometrics* **95**, 87–114 (2013).

[33] Cho, A. H. *et al.* Women are underrepresented on the editorial boards of journals in environmental biology and natural resource management. *PeerJ* **2**, e542 (2014).

[34] Erren, T. C., Groß, J. V., Shaw, D. M. & Selle, B. Representation of women as authors, reviewers, editors in chief, and editorial board members at 6 general medical journals in 2010 and 2011. *JAMA Intern. Med.* **174**, 633–635 (2014).

[35] Ioannidou, E. & Rosania, A. Under-representation of women on dental journal editorial boards. *PLoS One* **10**, e0116630 (2015).

[36] Metz, I., Harzing, A.-W. & Zyphur, M. J. Of journal editors and editorial boards: who are the trailblazers in increasing editorial board gender equality? *Br. J. Manag.* **27**, 712–726 (2016).

[37] Topaz, C. M. & Sen, S. Gender representation on journal editorial boards in the mathematical sciences. *PLoS One* **11**, e0161357 (2016).

[38] Dhanani, A. & Jones, M. J. Editorial boards of accounting journals: gender diversity and internationalisation. *Account. Audit. Account. J.* (2017).

[39] Gollins, C., Shipman, A. & Murrell, D. A study of the number of female editors-in-chief of dermatology journals. *Int. J. Womens. Dermatol.* **3**, 185–188 (2017).

[40] Piper, C. L., Scheel, J. R., Lee, C. I. & Forman, H. P. Representation of women on radiology journal editorial boards: a 40-year analysis. *Acad. Radiol.* **25**, 1640–1645 (2018).

[41] Fox, C. W., Duffy, M. A., Fairbairn, D. J. & Meyer, J. A. Gender diversity of editorial boards and gender differences in the peer review process at six journals of ecology and evolution. *Ecol. Evol.* **9**, 13636–13649 (2019).

[42] Hafeez, D. M. *et al.* Gender distribution in psychiatry journals' editorial boards worldwide. *Compr. Psychiatry* **94**, 152119 (2019).

[43] Harris, C. A. *et al.* Editorial (spring) board? Gender composition in high-impact general surgery journals over 20 years. *Ann. Surg.* **269**, 582 (2019).

[44] Jalilianhasanpour, R., Charkhchi, P., Mirbolouk, M. & Yousem, D. M. Underrepresentation of women on radiology editorial boards. *J. Am. Coll. Radiol.* **16**, 115–120 (2019).

[45] Kaji, A. H. *et al.* State of the journal: women first authors, peer reviewers, and editorial board members at annals of emergency medicine. *Ann. Emerg. Med.* **74**, 731–735 (2019).

[46] Litvack, J. R., Wick, E. H. & Whipple, M. E. Trends in female leadership at high-profile otolaryngology journals, 1997–2017. *Laryngoscope* **129**, 2031–2035 (2019).

[47] Lorello, G. R., Parmar, A. & Flexman, A. M. Representation of women on the editorial board of the canadian journal of anesthesia: a retrospective analysis from 1954 to 2018. *Can. J. Anaesth.* **66**, 989–990 (2019).

[48] Pagel, P. S., Freed, J. K. & Lien, C. A. Gender composition and trends of journal of cardiothoracic and vascular anesthesia editorial board membership: a 33-year analysis, 1987-2019. *J. Cardiothorac. Vasc. Anesth.* **33**, 3229–3234 (2019).

[49] Alonso-Arroyo, A., de Dios, J. G., Aleixandre-Agulló, J. & Aleixandre-Benavent, R. Gender inequalities on editorial boards of indexed pediatrics journals. *Pediatr. Res.* 1–15 (2020).

[50] Balasubramanian, S. *et al.* Women representation among cardiology journal editorial boards. *Circulation* **141**, 603–605 (2020).

[51] Rynecki, N. D., Krell, E. S., Potter, J. S., Ranpura, A. & Beebe, K. S. How well represented are women orthopaedic surgeons and residents on major orthopaedic editorial boards and publications? *Clin. Orthop. Relat. Res.* **478**, 1563–1568 (2020).

[52] Sarna, K. V. *et al.* Trends in gender composition on editorial boards in leading medicine, nursing, and pharmacy journals. *J. Am. Pharm. Assoc.* **60**, 565–570 (2020).

[53] Schurr, C., Müller, M. & Imhof, N. Who makes geographical knowledge? The gender of geography's gatekeepers. *Prof. Geogr.* **72**, 317–331 (2020).

[54] Alkhawtani, R. H., Kwee, T. C. & Kwee, R. M. Gender diversity among editorial boards of radiology-related journals. *Clin. Imaging* **75**, 30–33 (2021).

[55] Bennie, K. R. & Koka, S. Leadership diversity in prosthodontics: number and percentage of women chief editors of journals publishing prosthodontic science. *J. Prosthet. Dent.* (2021).

[56] Gottlieb, M. *et al.* Sex distribution of editorial board members among emergency medicine journals. *Ann. Emerg. Med.* **77**, 117–123 (2021).

[57] Hutchinson, D. *et al.* Emergency medicine journal editorial boards: analysis of gender, h-index, publications, academic rank, and leadership roles. *West. J. Emerg. Med.* **22**, 353 (2021).

[58] Pflibsen, L. R. *et al.* Representation of women on plastic surgery journal editorial boards in the united states. *Aesthet. Surg. J.* (2021).

[59] Pinho-Gomes, A.-C. *et al.* Representation of women among editors in chief of leading medical journals. *JAMA network open* **4**, e2123026–e2123026 (2021).

[60] Salazar, J. W., Claytor, J. D., Habib, A. R., Guduguntla, V. & Redberg, R. F. Gender, race, ethnicity, and sexual orientation of editors at leading medical and scientific journals: a cross-sectional survey. *JAMA Intern. Med.* (2021).

[61] Sperotto, R. A., Granada, C. E., Henriques, J. A. P., Timmers, L. F. S. & Contini, V. Editorial decision is still a men's task. *An. Acad. Bras. Cienc.* **93** (2021).

[62] Palser, E. R., Lazerwitz, M. & Fotopoulou, A. Gender and geographical disparity in editorial boards of journals in psychology and neuroscience. *Nature Neuroscience* **25**, 272–279 (2022).

[63] Wang, K. *et al.* Microsoft Academic Graph: When experts are not enough. *Quantitative Science Studies* **1**, 396–413 (2020).

[64] Wang, K. *et al.* A review of Microsoft Academic Services for Science of Science studies. *Frontiers in Big Data* **2**, 45 (2019).
