# Supplementary material for: Gender inequality and self-publication are common among academic editors
Source: Nat Hum Behav. 2023 Jan 16;7(3):353–64. doi: 10.1038/s41562-022-01498-1 (PMC10038799; doi:10.1038/s41562-022-01498-1)
Supplement: Supplementary file 2 — Reporting Summary [file 41562_2022_1498_MOESM2_ESM.pdf]

Corresponding author(s): Bedoor Alshebli
Talal Rahwan

Last updated by author(s): Nov 3, 2022

Reporting Summary

Nature Portfolio wishes to improve the reproducibility of the work that we publish. This form provides structure for consistency and transparency in reporting. For further information on Nature Portfolio policies, see our Editorial Policies and the Editorial Policy Checklist.

Statistics

For all statistical analyses, confirm that the following items are present in the figure legend, table legend, main text, or Methods section.

n/a	Confirmed	
☐	☒	The exact sample size (*n*) for each experimental group/condition, given as a discrete number and unit of measurement
☐	☒	A statement on whether measurements were taken from distinct samples or whether the same sample was measured repeatedly
☐	☒	The statistical test(s) used AND whether they are one- or two-sided<br>*Only common tests should be described solely by name; describe more complex techniques in the Methods section.*
☐	☒	A description of all covariates tested
☐	☒	A description of any assumptions or corrections, such as tests of normality and adjustment for multiple comparisons
☐	☒	A full description of the statistical parameters including central tendency (e.g. means) or other basic estimates (e.g. regression coefficient) AND variation (e.g. standard deviation) or associated estimates of uncertainty (e.g. confidence intervals)
☐	☒	For null hypothesis testing, the test statistic (e.g. *F*, *t*, *r*) with confidence intervals, effect sizes, degrees of freedom and *P* value noted<br>*Give P values as exact values whenever suitable.*
☒	☐	For Bayesian analysis, information on the choice of priors and Markov chain Monte Carlo settings
☒	☐	For hierarchical and complex designs, identification of the appropriate level for tests and full reporting of outcomes
☒	☐	Estimates of effect sizes (e.g. Cohen's *d*, Pearson's *r*), indicating how they were calculated

Our web collection on statistics for biologists contains articles on many of the points above.

Software and code

Policy information about availability of computer code

Data collection	Data about editors was collected using ElSevier's Article Retrieval API: https://dev.elsevier.com/documentation/ArticleRetrievalAPI.wadl<br>Data about the publication records of individual scientists was retrieved from the Microsoft Academic Graph website.<br>The gender of the authors in the dataset, was collected from the API of Genderize.io.<br>The code used to collect and clean data can be found in our Github repository (https://github.com/Michael98Liu/fair-and-inclusive-scientific-publishing/tree/main/data_collection).
Data analysis	Data analysis was done using computer programs written in the Python programming language (version 3.8.5). You can find the code in our Github repository (https://github.com/Michael98Liu/fair-and-inclusive-scientific-publishing/).

For manuscripts utilizing custom algorithms or software that are central to the research but not yet described in published literature, software must be made available to editors and reviewers. We strongly encourage code deposition in a community repository (e.g. GitHub). See the Nature Portfolio guidelines for submitting code & software for further information.

Data

Policy information about availability of data

All manuscripts must include a data availability statement. This statement should provide the following information, where applicable:

- Accession codes, unique identifiers, or web links for publicly available datasets
- A description of any restrictions on data availability
- For clinical datasets or third party data, please ensure that the statement adheres to our policy

Our editors' dataset was collected from Elsevier's ScienceDirect database. A formal agreement between us and Elservier mandates that data copied from the subscribed products cannot be provided to third parties in any substantial or systematic manner. However, for transparency reasons, we provide a sample set of 10 editors, which can be used to test the code for data collection and analysis, along with anonymized data for reproducing figures, all the while ensuring that our agreement with Elsevier is not breached. As for our publications' dataset, i.e., the Microsoft Academic Graph, a copy of it is available at https://zenodo.org/record/2628216#.Yx9BbexBza4; a small subset of MAG that is sufficient to test our code is also provided. To retrieve the aforementioned datasets, visit https://github.com/Michael98Liu/fair-and-inclusive-scientific-publishing/tree/main/data.

Human research participants

Policy information about studies involving human research participants and Sex and Gender in Research.

Reporting on sex and gender	There were no participants involved.
Population characteristics	See above.
Recruitment	There were no participants involved.
Ethics oversight	There were no participants involved.

Note that full information on the approval of the study protocol must also be provided in the manuscript.

Field-specific reporting

Please select the one below that is the best fit for your research. If you are not sure, read the appropriate sections before making your selection.

☐ Life sciences ☒ Behavioural & social sciences ☐ Ecological, evolutionary & environmental sciences

For a reference copy of the document with all sections, see nature.com/documents/nr-reporting-summary-flat.pdf

Behavioural & social sciences study design

All studies must disclose on these points even when the disclosure is negative.

Study description	We use matching techniques to study the relationship between editors assuming their roles and their publication patterns.<br>We used randomized baseline models to understand the relationship between the population of female scientists and female editors. The data is quantitative.
Research sample	We focus on all Elsevier journals, and study all editors whose name can be automatically retrieved from Elsevier database using the aforementioned computer program and API. Data is collected using computer program, and therefore demographics information is not available. The sample of editors that we study is not meant to be a representative sample of all editors in the world. We choose to study Elsevier because it is one of the largest publishers, by generating one fifth of global research output, and garnering one quarter of citations worldwide.<br><br>This study also uses an existing dataset called the Microsoft Academic Graph (MAG, source: https://www.microsoft.com/en-us/research/project/microsoft-academic-graph/).
Sampling strategy	We use convenience sampling. Our sample consists of all those whose name can be automatically extracted from Elsevier database. No sample-size calculation was performed; rather, we study all 103,000 editors whose name is identifiable. These data is sufficient since they cover 15 different scientific disciplines, and span over four decades.
Data collection	The publication dataset was downloaded from Microsoft Academic Graph's website.<br>The editors dataset was collected using Elsevier's Article Retrieval API.<br>There were no participants involved; researchers were aware of the research hypothesis during data collection.
Timing	The data was collected in January 2020.

Data exclusions	In order to perform gender-related analysis, we identify the gender 81,000 editors with at least 90% accuracy. The remaining ones were excluded from our gender-based analysis. This exclusion criteria was pre-determined, following past studies.<br><br>In order to perform bibliometrics analysis, we were able to identify the publication records of 20,000 editors. The remaining ones were excluded from our bibliometrics analysis. This exclusion criteria was pre-determined to avoid the arduous task of manually identifying editors from over 200 million scientists recorded in MAG.<br><br>For more discussion regarding the mentioned data exclusion, please see the "Dataset Evaluation" subsection in the Methods section of our main manuscript.
Non-participation	There were no participants involved.
Randomization	There were no participants involved.

Reporting for specific materials, systems and methods

We require information from authors about some types of materials, experimental systems and methods used in many studies. Here, indicate whether each material, system or method listed is relevant to your study. If you are not sure if a list item applies to your research, read the appropriate section before selecting a response.

Materials & experimental systems

n/a	Involved in the study
☒	☐ Antibodies
☒	☐ Eukaryotic cell lines
☒	☐ Palaeontology and archaeology
☒	☐ Animals and other organisms
☒	☐ Clinical data
☒	☐ Dual use research of concern

Methods

n/a	Involved in the study
☒	☐ ChIP-seq
☒	☐ Flow cytometry
☒	☐ MRI-based neuroimaging
